# Systemic administration of glucocorticoids, cardiovascular complications and mortality in patients hospitalised with COVID-19, SARS, MERS or influenza: A systematic review and meta-analysis of randomised trials

**DOI:** 10.1016/j.phrs.2021.106053

**Published:** 2022-02

**Authors:** Elisabetta Caiazzo, Asma O.M. Rezig, Dario Bruzzese, Armando Ialenti, Carla Cicala, John G.F. Cleland, Tomasz J. Guzik, Pasquale Maffia, Pierpaolo Pellicori

**Affiliations:** a Centre for Immunobiology, Institute of Infection, Immunity and Inflammation, College of Medical, Veterinary and Life Sciences, University of Glasgow, UK; b Department of Pharmacy, School of Medicine and Surgery, University of Naples Federico II, Naples, Italy; c Department of Public health, University of Naples Federico II, Naples, Italy; d Robertson Centre for Biostatistics, and Glasgow Clinical Trials Unit, Institute of Health and Wellbeing, University of Glasgow, UK; e National Heart & Lung Institute, Imperial College London, UK; f Institute of Cardiovascular and Medical Sciences, College of Medical, Veterinary and Life Sciences, University of Glasgow, UK; g Department of Internal and Agricultural Medicine, Jagiellonian University Medical College, Krakow, Poland

**Keywords:** COVID-19, Glucocorticoids, Steroids, Dexamethasone, Mortality, Meta-analysis

## Abstract

**Background:**

Administration of glucocorticoids might reduce mortality in patients with severe COVID-19 but have adverse cardiometabolic effects.

**Objectives:**

to investigate the effect of systemic administration of glucocorticoids on cardiovascular complications and all-cause mortality in patients hospitalised with respiratory viral infections, including COVID-19, SARS, MERS and influenza.

**Methods:**

We identified randomised trials published prior to July 28th, 2021. The Mantel-Haenszel random effects method and the Hartung and Knapp adjustment were used to obtain pooled estimates of treatment effect with 95% confidence intervals.

**Results:**

No randomised trials of glucocorticoids for SARS, MERS or influenza reported relevant outcomes. We included eleven COVID-19 randomised trials (8109 patients). Overall, compared to placebo or standard care, glucocorticoids were not associated with a reduction of in-hospital mortality (p = 0.09). In a pre-specified sub-analysis, in-hospital mortality was reduced by 19% when follow-up was restricted to 14 days from randomisation (5/11 trials, 1329 patients, p = 0.02). With longer follow-up (9/11 trials, 7874 patients), administration of glucocorticoids was associated with a trend to benefit for those requiring mechanical ventilation (RR 0.86; 95% CI 0.57–1.27) but possible harm for those not receiving oxygen at randomisation (RR 1.27; 95% CI 1.00 – 1.61), an effect that was significantly different amongst subgroups (p = 0.0359). Glucocorticoids reduced the risk of worsening renal function by 37% (4/11 trials); reported rate of other cardiovascular complications was low.

**Conclusions:**

Administration of systemic glucocorticoids to patients hospitalised with COVID-19 does not lower mortality overall but may reduce it in those requiring respiratory support and increase it in those who do not.

## Graphical Abstract

**Figure:**
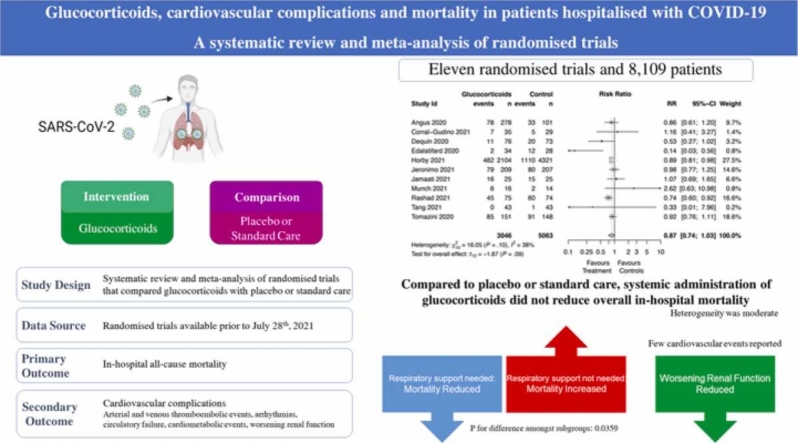

## 1 Introduction

Glucocorticoids are potent anti-inflammatory drugs that have been used, without much supporting evidence, as adjuvant therapy for viral pneumonias, including severe acute respiratory syndrome (SARS) [1], Middle East respiratory syndrome (MERS) [2] and influenza [3], but have now been more widely studied in patients with COVID-19. In the largest trial, RECOVERY [4], a ten-day course of dexamethasone was associated with a survival benefit in patients hospitalised with COVID-19, especially amongst those requiring ventilation or oxygen therapy; these findings were supported by a meta-analysis that included an additional six trials, leading to dexamethasone rapidly being recommended in therapeutic protocols for patients with severe COVID-19 [5].

However, the mechanisms underlying the association between improved survival and use of glucocorticoids in critically ill patients with COVID-19 are not fully understood. Subsets of patients who might not benefit or be harmed may also exist [4]. Cardiovascular complications such arrhythmias, heart failure and arterial or venous thromboembolic events are common in patients with a severe COVID-19 infection [6], [7], [8]. Glucocorticoids can have adverse metabolic effects, including hyperglycaemia and salt and water retention [9], [10], [11], [12], which could lead to development of hypertension, arrhythmias, worsening diabetes and heart failure. It is unclear to what extent the potential benefits of glucocorticoids are outweighed by their potential harms.

It is also unclear if the beneficial effects of glucocorticoids on mortality are specific for COVID-19 or apply to other viral respiratory infections. Therefore, we conducted a systematic review and meta-analysis to assess the association between the use of glucocorticoids with cardiovascular complications and mortality in randomised trials that enrolled adults hospitalised with a suspected or confirmed infection caused by SARS-CoV-2, other highly pathogenic and deadly human coronaviruses (MERS, SARS) or influenza, to elucidate further their safety and effectiveness in these populations.

## 2 Methods

The protocol of this systematic review and meta-analysis is registered on PROSPERO (CRD42021270992). This study followed the Preferred Reporting Items for Systematic Reviews and Metaanalyses (PRISMA) reporting guideline.

### 2.1 Data sources and search strategy

We systematically searched for pre-prints and published peer-reviewed studies in Medline (PubMed), Embase (Ovid), the Cochrane Central Register of Controlled Trials (CENTRAL), and the Cochrane COVID-19 register to identify randomised trials that evaluated the use of glucocorticoids in patients with COVID-19, MERS, SARS and influenza. The search strategy consisted of the definition of condition, the intervention applied and the study design including Medical Subject Headings (MeSH) terms and using keyword search limited to the title and abstract. The detailed search strategy is shown in the Supplementary material. EC and AOMR, informed by two content experts (PP and PM), drafted a search strategy; the search was conducted on 28 July 2021 by EC and AOMR, guided by an experienced librarian of the University of Glasgow, Paul Cannon.

### 2.2 Eligibility

We only included peer-reviewed or pre-print randomised trials that enrolled adults (age ≥ 18 years) hospitalised with suspected or confirmed respiratory viral infections including SARS-CoV-2, SARS, MERS, and influenza, which compared any dose or type of glucocorticoids given orally or intravenously, regardless of the duration of treatment, with a placebo or standard care group. Trials comparing only different types or doses of glucocorticoids were excluded, as were observational studies, editorials, letters, reviews, guidelines, case studies, case reports, case series, or cross-sectional studies, abstracts and conference proceedings, systematic reviews and meta-analyses, ongoing studies and articles that were not available in English.

### 2.3 Definition of primary and secondary outcomes

The primary outcome was all-cause mortality at the longest follow-up duration. Follow-up between baseline and 14-days and between baseline and 21–28 days was also considered. The secondary outcomes were the incidence of cardiovascular and renal events, including arterial events (myocardial infarction and stroke), venous events (deep vein thrombosis and pulmonary embolism), arrhythmias (supraventricular and ventricular arrhythmias), circulatory failure (shock, need for vasopressor therapy or heart failure), cardiometabolic events (hyperglycaemia or need for insulin therapy), and worsening renal function.

### 2.4 Study selection

Included studies were selected in two stages. Initially, two review authors (EC, AOMR) independently screened all studies by title and abstract, then by full texts. Studies were only included if there was agreement between the two authors.

### 2.5 Data extraction and risk of bias

After selection, data were independently extracted by two review authors (EC, AOMR). Where available, for each study, we collected:

• Study design, size and countries where the randomised trial was conducted.
• Baseline disease characteristics, settings and severity (i.e.: need for invasive mechanical ventilation, need for intensive care unit admission, need for vasopressor therapy, partial pressure of arterial oxygen to the fraction of inspired oxygen ratio (PaO2:FiO2), and days from symptom onset to randomisation or hospitalization).
• Participants’ baseline demographics (including age, gender, ethnicity, systolic/diastolic blood pressure and body mass index).
• Participants’ baseline comorbidities (including diabetes mellitus, hypertension, coronary artery disease or cardiovascular disease, heart failure, asthma, chronic obstructive pulmonary disease, history of tuberculosis, chronic kidney disease, anaemia, history of cancer, and obesity).

Two review authors (EC, AOMR) independently assessed the quality of evidence using the Grades of Recommendation, Assessment, Development and Evaluation (GRADE) approach. They also assessed the risk of bias on five domains (randomisation process, deviations from the intended interventions, missing outcome data, measurement of the outcome, and selection of the reported result) using the Cochrane Risk of Bias 2.0 (RoB2) tool. Any disagreements in study selection, data extraction, or quality of evidence assessment were escalated to a third review author (PP) who helped in reaching a consensus.

### 2.6 Statistical analysis

The Mantel-Haenszel random effects method was used to obtain pooled estimates of treatment effect (Risk Ratio - RR) with the associated 95% confidence interval (CI). The DerSimonian-Laird method for estimating the between-study variance t^2^ was used. Due to the small number of trials included, the Hartung and Knapp (HK) adjustment was employed. Heterogeneity was firstly assessed through visual inspection of the forest plots, and then estimated using the *I*^*2*^ statistic: we considered an *I*^*2*^ of 25%, 26–50%, and > 50% as low, moderate, and high heterogeneity, respectively. Sensitivity analyses were conducted to assess for robustness of synthesised results and to assess for the impact of including trials at all risks of bias. Subgroup analyses were conducted to investigate the impact of disease severity at randomisation and treatment effect interactions. Publication bias was visually examined using funnel plot (eFigure1 in the Supplement). Statistical analysis was performed using the R statistical programming environment, Version 4.02 (http://www.r-project.org). Package meta, Version 4.13 [13] was used for all the meta-analysis elaborations.

## 3 Results

### 3.1 Search results

After removing duplicates, we retrieved 4558 records from Embase (Ovid), Medline (PubMed), CENTRAL and the COVID-19 study registry to be screened for inclusion; of these, 321 full-text articles were examined for eligibility. We finally included 11 randomised trials in our meta-analysis (PRISMA Flow Diagram shown below); a large proportion of those excluded were observational studies, editorials, reviews or case reports. We did not identify any randomised trial assessing the use of glucocorticoids in adult patients with suspected or confirmed MERS. We identified two randomised trials assessing the use of prednisone in community-acquired pneumonia, including viral influenza [14], [15]. Of 785 randomised patients, Blum and colleagues [14] reported 27 patients with laboratory confirmed influenza but outcome data were not assessed separately for this subgroup. Of 726 randomised patients, Wirz and colleagues [15] identified 24 with laboratory confirmed influenza; of these, one patient assigned to prednisone and one to placebo died. Therefore, we did not include these trials in our meta-analysis. We retrieved one randomised trial [16] assessing the effect of early hydrocortisone administration on SARS-CoV RNA levels in 16 patients, but it was excluded as it did not report any other relevant outcome measures. Two randomised trials, by Ranjbar et al. [17] and Munch et al. [18] were also excluded as they compared the efficacy of two active interventions, methylprednisolone and dexamethasone, or two doses of dexamethasone, respectively, in patients with COVID-19 but without a group assigned to placebo or standard care.

PRISMA Flow Diagram for Search: The flow diagram was created in consort with PRISMA guidelines [60].

### 3.2 Description of included trials

We included 11 randomised trials that enrolled 8109 patients (Table 1). Eight randomised trials [4], [19], [20], [21], [22], [23], [24], [25] were multi-centre, with RECOVERY [4] contributing 80% of all patients to the meta-analysis. The remaining trials [26], [27], [28] were conducted in single sites in Iran, Brazil, and Egypt.

**Table 1:** General characteristics of included trials.

Trial	Patients (Intervention: Control)	Intervention	Control	Main Inclusion Criteria	Main Exclusion Criteria	Primary Outcomes
**Angus 2020 REMAP-CAP** (International) [19]	384 283:101	Hydrocortisone (IV) 50 mg QID for 7days; or shock dependent dose for up to 28 days.	Standard Care	Hospitalised	> 14 days since hospitalization or > 36 h since ICU admission.	Resp. and CV support-free days up to day 21.
**Corral-Gudino 2021** **GLUCOCOVID** (Spain) [20]	64 35:29	Methylprednisolone (IV) (80 mg for 3 days, then 40 mg for 3 days)	Standard Care	Hospitalised with symptom duration of ≥ 7 days, elevated systemic inflammatory biomarkers.	Mechanically ventilated, ICU, CKD on dialysis, pregnancy.	Composite (in hospital all-cause mortality, ICU admission or progression of respiratory insufficiency).
**Dequin 2020** **CAPECOVID** (France) [21]	149 76:73	Hydrocortisone (IV) (200 mg/day for 7 days, then 100 mg/day for 4 days and 50 mg/day for 3 days)	Placebo	Hospitalised with acute respiratory failure	Vasopressor for septic shock at baseline, other viral pneumonia or active lung infection, pregnancy.	Treatment failure on day 21.
**Edalatifard 2020** (Iran) [22]	62 34:28	Methylprednisolone (IV) (250 mg/day for 3 days)	Standard Care	Hospitalised SpO_2_ < 90%, elevated CRP and IL-6, no ventilatory support.	Pregnancy, ARDS, uncontrolled HTN or DM, HF, immunosuppressive drugs.	Time of clinical improvement and time of hospital discharge or death.
**Horby 2021** **RECOVERY** (United Kingdom) [4]	6425 2104:4321	Dexamethasone (Oral/IV) (6 mg/day for 10 days)	Standard Care	Hospitalised	Contraindication to dexamethasone.	28 days all-cause mortality.
**Jamaati 2021** (Iran) [26]	50 25:25	Dexamethasone (IV) (20 mg/day from day 1–5 then 10 mg/day until day 10)	Standard Care	Hospitalised PaO_2_/FiO_2_ between 100 and 300.	CKD, chronic liver disease, hyperglycaemia, pregnancy	Need for IMV and death rate.
**Jeronimo 2020** **Metcovid** (Brazil) [27]	416 209:207	Methylprednisolone (IV) (1 mg/kg for 5 days)	Placebo	Hospitalised	CKD, decompensated cirrhosis, HIV/AIDS, pregnancy/breastfeeding.	28-day mortality.
**Munch 2021** **COVID STEROID** (Denmark) [23]	30 16:14	Hydrocortisone (IV) (200 mg/day for 7 days)	Placebo	Hospitalised	IMV for > 48 h prior to screening, pregnancy.	Days alive without use of life support at day 28.
**Rashad 2021** (Egypt) [28]	109 63:46	Dexamethasone (IV) (Infusion 4 mg/kg/day for 3 days, then 8 mg/day for 10 days)	Tocilizumab	Hospitalised and elevated inflammatory biomarkers.	Active bacterial or fungal infections, interstitial lung disease, death before third day of ICU admission.	Mortality within 14 days from ICU admission.
**Tang 2021** (China) [24]	86 43:43	Methylprednisolone (IV) (1 mg/kg/day for 7 days)	Placebo	Hospitalised < 72 h.	Severe immunosuppression, refractory HTN or hypokalaemia, secondary bacterial/fungal infections.	Clinical cure at 14 days.
**Tomazini 2020** **CoDEX** (Brazil) [25]	299 151:148	Dexamethasone (IV) (20 mg/day for 5 days, then 10 mg/day for 5 days)	Standard Care	Adults intubated and mechanically ventilated	Pregnancy, use of immunosuppressive drugs	Ventilator-free days during the first 28 days.

ICU: intensive care unit, IMV: invasive mechanical ventilation, CKD: chronic kidney disease, PaO_2_: FiO_2_ partial pressure of arterial oxygen to the fraction of inspired oxygen ratio, SpO_2_: oxygen saturation, CRP: C-reactive protein, IL-6: interleukin-6, ARDS: acute respiratory distress syndrome, HTN: hypertension, DM: diabetes mellitus, HF: heart failure, HIV: human immunodeficiency virus, AIDS: acquired immune deficiency syndrome, QID: four times daily, CV: cardiovascular, Resp. respiratory.

Five trials [19], [20], [21], [23], [25] were discontinued or terminated early following the positive report from the RECOVERY trial [4]. The trial led by Tang and colleagues [24] was terminated early due to slow recruitment.

Five trials [4], [19], [21], [25], [27], including RECOVERY, enrolled patients with suspected or laboratory confirmed COVID-19. Six trials [20], [22], [23], [24], [26], [28] only included patients with confirmed SARS-CoV-2 by PCR testing. Interventions varied across randomised trials: three trials evaluated hydrocortisone [19], [21], [23], four dexamethasone [4], [25], [26], [28], and four methylprednisolone [20], [22], [24], [27]. The control group consisted of standard care alone in six trials, including RECOVERY [4], [19], [20], [22], [25], [26], placebo in four trials [21], [23], [24], [27] and an active comparator, tocilizumab, in Rashad et al. [28]. The main characteristics of included trials are summarised in Table 1.

### 3.3 Baseline characteristics of included trials

Overall, 89% of participants were reported to have had a positive SARS-CoV-2 PCR test (Table 2). The median age of patients enrolled ranged from 55 to 70 years, and they were more likely to be men (64%). About half of patients had a history of hypertension, 1 in 4 had diabetes, 1 in 4 had cardiovascular disease, 1 in 5 had chronic respiratory disease and less than 10% had chronic kidney disease. When reported, oxygen therapy or non-invasive ventilation was required in 59% and mechanical ventilation in 23% at the time of randomisation.

**Table 2:** Baseline characteristics of included trials.

Variable n/N (%)	Angus 2020 REMAPCAP	Corral-Gudino 2021 GLUCO COVID	Dequin 2020 CAPE COVID	Edalatifard 2020	Horby 2021 RECOVERY	Jamaati 2021	Jeronimo 2021 Metcovid	Munch 2021 COVID STEROID	Rashad 2021	Tang 2021	Tomazini 2020 CoDEX
**SARS-CoV-2**	275/359 (77)	64/64 (100)	144/149 (97)	62/62 (100)	5744/6425 (89)	50/50 (100)	318/391 (81)	30/30 (100)	109/109 (100)	86/86 (100)	286/299 (96)
**Age**^a^	60 ± 13	70 ± 12	62 ± 13	59 ± 17	66 ± 16	~62	55 ± 15	61 (53, 73)	~62	56 (39, 66)	61 ± 14
**Sex** **(Female)**	111/384 (29)	25/64 (39)	45/149 (30)	23/62 (37)	2338/6425 (36)	14/50 (28)	144/415 (35)	6/30 (20)	47/109 (43)	45/86 (52)	112/299 (38)
**Diabetes Mellitus**	119/371 (32)	11/64 (17)	27/149 (18)	22/62 (35)	1546/6425 (24)	27/50 (54)	111/382 (29)	4/30 (13)	31/109 (28)	8/86 (9)	126/299 (42)
**HTN**	NA	30/64 (47)	NA	20/62 (32)	NA	25/50 (50)	185/382 (48)	8/30 (27)	52/109 (48)	31/86 (36)	198/299 (66)
**Heart Disease**^b^	28/375 (7)	8/64 (13)	NA	11/62 (18)	1757/6425 (27)	7/50 (14)	25/381 (7)	1/30 (3)	14/109 (13)	6/86 (7)	NA
**Respiratory Disease**^c^	75/369 (20)	5/64 (8)	11/149 (7)	6/62 (10)	1371/6425 (21)	10/50 (20)	19/382 (5)	5/30 (17)	5/109 (5)	5/86 (6)	NA
**Heart Failure**	NA	NA	6/94 (6)	0/62 (0)	NA	NA	NA	1/30 (3)	NA	NA	23/299 (8)
**CKD**	32/347 (9)	NA	4/94 (4)	7/62 (11)	524/6425 (8)	0/50 (0)	0/416 (0)	1/30 (3)	9/109 (8)	1/86 (1)	16/299 (5)
**IMV**	213/384 (55)	0/64 (0)	121/149 (81)	0/62 (0)	1007/6425 (16)	NA	141/416 (34)	11/30 (37)	39/109 (36)	NA	299/299 (100)
**Oxygen or** **NIV**	170/384 (44)	64/64 (100)	28/149 (19)	62/62 (100)	3883/6425 (60)	50/50 (100)	197/415 (47)	19/30 (63)	70/109 (64)	61/86 (71)	NA
**No Oxygen**	1/384 (<1)	0/64 (0)	0/149 (0)	0/62 (0)	1535/6425 (24)	0/50 (0)	NA	0/30 (0)	0/109 (0)	NA	NA
**ICU Admission**	384/384 (100)	0/64 (0)	149/149 (100)	NA	NA	NA	131/370 (35)	22/30 (73)	109/109 (100)	0/86 (0)	299/299 (100)
**Vasopressor Use**	133/384 (35)	NA	31/149 (21)	NA	NA	NA	NA	10/30 (33)	55/109 (50)	NA	200/299 (67)

a Age measured in years. Values represent mean (standard deviation) or median (interquartile range) for the total population unless otherwise specified.

b Heart disease includes coronary artery disease and heart failure.

c Respiratory disease includes chronic obstructive pulmonary disease, asthma, and history of tuberculosis. SARS-CoV-2: severe acute respiratory syndrome coronavirus 2, HTN: hypertension, CKD: chronic kidney disease, IVM: invasive mechanical ventilation, NIV: non-invasive ventilation, ICU: intensive care unit.

### 3.4 Risk of bias in included trials and assessment of outcomes

Risk of bias was assessed for the outcomes of in-hospital mortality and worsening renal function. Five randomised trials [4], [19], [21], [23], [27] were assessed at low risk of bias, five with some concerns [20], [22], [24], [25], [26] and one [28] with high risk of bias (eTable1 and eTable2 in the Supplement). All trials were included in the meta-analysis with sensitivity analyses excluding [28] (eFigure2 in the Supplement).

#### 3.4.1 In-hospital mortality

**Fig. 1:**
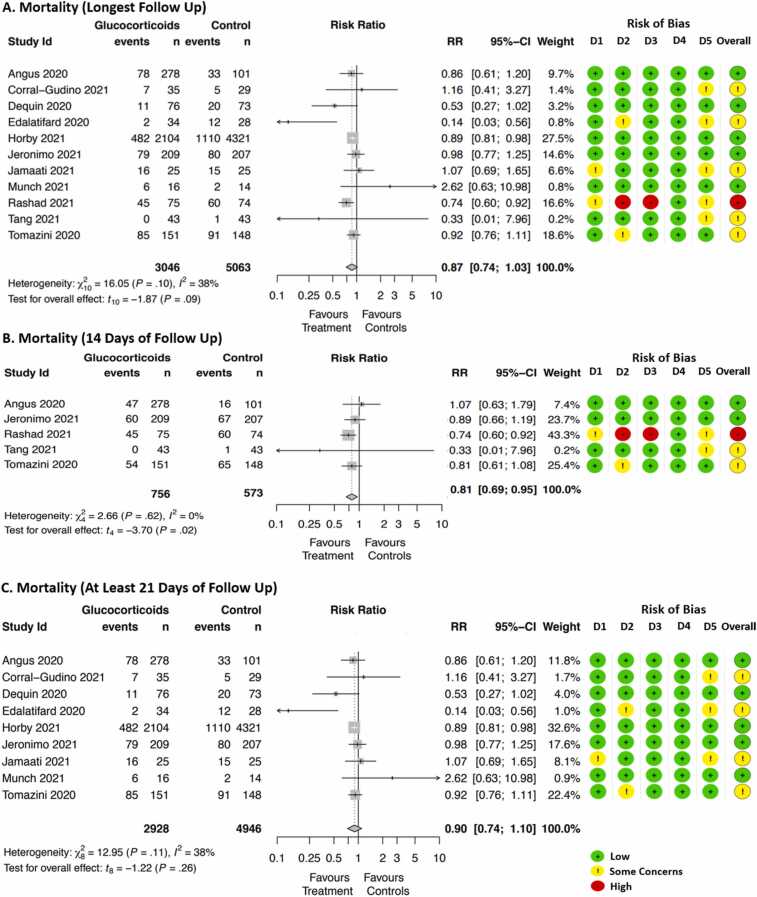
Effect of glucocorticoids on in-hospital mortality in COVID-19 patients. (A) longest follow up, (B) 14-days and (C) at least 21 days of follow up. Grey coloured squares show the effect estimate (risk ratios) with the size of each square corresponding to the weight given to each trial in the meta-analysis. Horizontal lines represent the 95% CIs corresponding to each effect estimate. The diamond represents the overall effect of intervention with its width representing the overall 95% CI. The I^2^ statistic represents a measure of heterogeneity. Risk of bias is reported for each trial assessing five domains: (D1) Randomisation process, (D2) Deviations from the intended interventions, (D3) Missing outcome data, (D4) Measurement of the outcome, (D5) Selection of the reported result.

Nine trials reported follow-up for at least 21 days and up to 28 days, three of which [19], [25], [27] also reported rate of deaths at 14 days; Tang [24] and Rashad [28] only evaluated mortality at 14 days (eTable3 in the Supplement). Due to heterogeneity on follow-up duration across included trials, we first assessed in-hospital mortality based on the longest available follow-up duration. A pooled analysis suggested a 13% reduction in mortality associated with administration of glucocorticoids compared to standard care or placebo (RR 0.87; 95%CI: 0.74 – 1.03, p = 0.09) (Fig. 1A and graphical abstract), which did not reach statistical significance, but a 19% reduction (RR 0.81; 95% CI: 0.69 – 0.95, p = 0.02) when the analysis was restricted to 14 days from randomisation (5/11 trials, 1329 patients) (Fig. 1B). However, for trials with follow-up of at least 21 days, which included the RECOVERY trial, the overall reduction in mortality was insignificant (RR 0.90; 95% CI: 0.74 – 1.10, p = 0.26) (Fig. 1C).

**Fig. 2:**
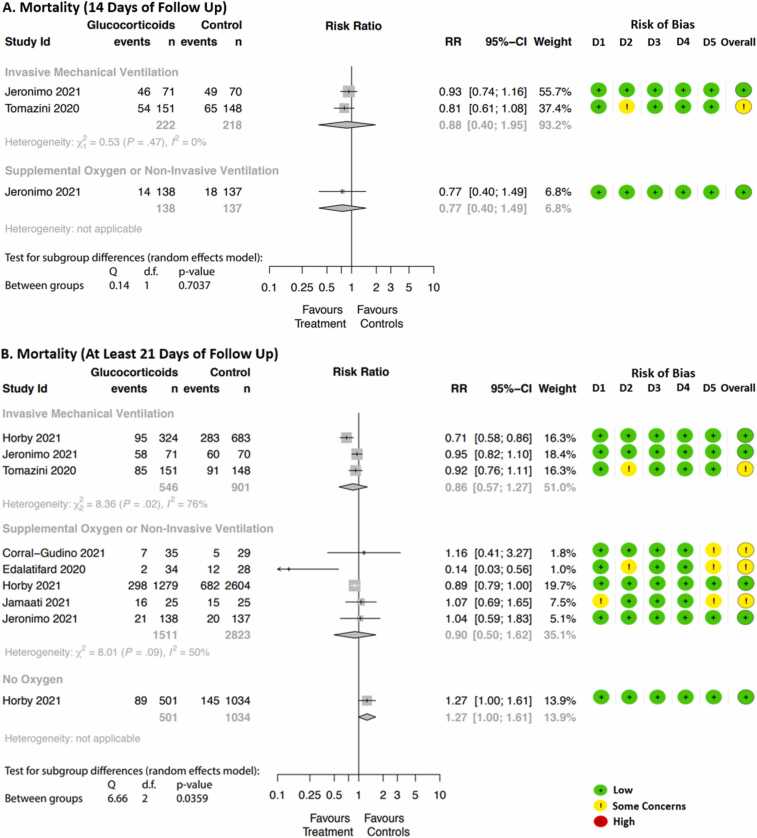
Subgroup analysis of in-hospital mortality by severity at randomisation. (A) at 14 days of follow up and (B) at least 21 days of follow up**.** Grey-coloured squares show the effect estimate (risk ratios) with the size of each square corresponding to the weight given to each study in the meta-analysis. Horizontal lines represent the 95% CIs corresponding to each effect estimate. The diamond is an estimate of the overall effect of intervention with its width representing the overall 95% CI. The I^2^ statistic represents a measure of heterogeneity. Risk of bias is reported for each trial assessing five domains: (D1) Randomisation process, (D2) Deviations from the intended interventions, (D3) Missing outcome data, (D4) Measurement of the outcome, (D5) Selection of the reported result.

For the 14-days outcome, comparisons between patients requiring invasive mechanical ventilation (n = 440) at randomisation and those requiring oxygen support (n = 275) did not show any difference in mortality associated with glucocorticoid use (Fig. 2 A, p = 0.70). For trials with at least 21 days of follow-up, numbers were larger (1447 and 4334 respectively) but again, there was no clear evidence of benefit from administration of glucocorticoids. However, there was evidence of harm for those not receiving oxygen at randomisation (n = 1535; 27% increased risk in mortality if assigned to glucocorticoids) but a trend to benefit in those requiring respiratory support, an effect that was significantly different amongst subgroups (Fig. 2B, p = 0.0359). We did not find an effect interaction with type of glucocorticoid used (eFigure3 in the Supplement) or type of control treatment (eFigure4 in the Supplement). Using the GRADE assessment tool, we report moderate certainty of our findings due to inclusion of one trial [28] at high risk of bias that contributed substantially to the weighted meta-analysis risk estimate (16.6% and 43.3% to the outcomes of in-hospital mortality at the longest follow up duration and at 14 days, respectively).

#### 3.4.2 Cardiovascular and renal outcomes

We found a low incidence of cardiovascular events (eTable3 in the Supplement). To quantify the extent of under-reporting, we estimated the expected rate of cardiovascular events in patients with COVID-19 using a recent systematic review of prevalence studies as reference 6. Among all secondary outcomes, only incidence of worsening renal function (5.8%; reported in four trials) was similar to that reported by Pellicori et al. (5.1%) (eTable4 in the Supplement), whilst incidence of other events was substantially lower, up to 100 times lower for pulmonary embolism and DVT.

**Fig. 3:**
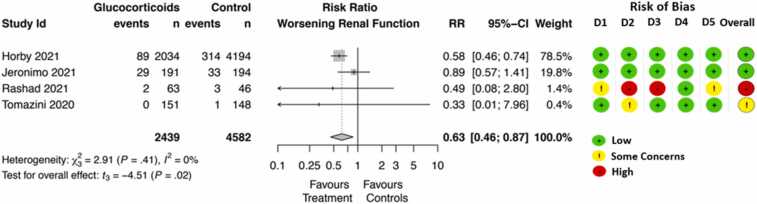
Effect of glucocorticoids on worsening renal function in hospitalized COVID-19 patients. Grey-coloured squares show the effect estimate (risk ratios) with the size of each square corresponding to the weight given to each study in the meta-analysis. Horizontal lines represent the 95% CIs corresponding to each effect estimate. The diamond represents the overall effect of intervention with its width representing the overall 95% CI. The I2 statistic is a measure of heterogeneity. Risk of bias is reported for each trial assessing five domains: (D1) Randomisation process, (D2) Deviations from the intended interventions, (D3) Missing outcome data, (D4) Measurement of the outcome, (D5) Selection of the reported result.

We found that, compared to other treatments or placebo, administration of glucocorticoids was associated with a 37% reduction of worsening renal function (RR 0.63; 95% CI 0.46 – 0.87, p = 0.02) (Fig. 3) and a high certainty of evidence. Compared to other interventions or placebo, we found no evidence that glucocorticoids influenced a broad range of cardiovascular events (eFigure5 in the Supplement).

## 4 Discussion

Glucocorticoids are often administered to patients with respiratory viral infections other than COVID-19 [2], [3], but their effectiveness is controversial. Meta-analyses of observational studies suggested possible harm for patients with influenza [29], [30], [31], [32] or SARS [33]. Many experts were concerned about steroid-induced immunosuppression, reducing viral clearance and predisposing to secondary bacterial infection [34], [35], and the effects of steroids on glucose, salt and water metabolism, particularly for patients with heart failure and/or diabetes.

In this systematic review and meta-analysis, we found that administration of glucocorticoids did not significantly reduce mortality for patients hospitalised with COVID-19 overall. However, glucocorticoids may have reduced mortality for sicker patients requiring respiratory support but increased mortality amongst those who did not. This is fairly consistent with the findings of the RECOVERY trial [4], which reported a reduction in mortality with the administration of glucocorticoids only amongst those hospitalised with COVID-19 who required respiratory support. Importantly, our analysis suggests that the non-significant trend observed in RECOVERY, for a worse outcome with dexamethasone for patients who did not require respiratory support, is real. Physicians might be tempted to administer steroids prior to the need for respiratory support in the absence of evidence of harm. This analysis suggests that a more conservative approach is warranted, because the harm may outweigh any benefit when steroids are given to such patients. However, there must be some patients where the benefits and harm of steroids are balanced. Whether steroids should be withheld when oxygen therapy alone suffices to correct hypoxaemia deserves further investigation as does their use in patients on the verge of needing, but not yet receiving, respiratory support. Should steroids perhaps be withheld until ventilation, non-invasive or invasive, is required to maintain oxygenation? However, it is also possible that the amount of respiratory support just reflects disease severity, and that oxygenation is not a good measure of the need for steroids.

There are many mechanisms by which glucocorticoids might have favourable effects in some patients hospitalised with COVID-19. Uncertainty exists on the extent to which organ damage reflects COVID-19 viral infection itself or the immune response to the virus [36]. Autopsy reports suggest that SARS-CoV-2 may cause endothelial inflammation, directly or by immune activation, leading to micro-vascular dysfunction and occlusion in the lungs and other organs. An exaggerated pulmonary and systemic immune response might be a key pathophysiological mechanism driving poor outcomes in many patients with COVID-19, the modulation of which by glucocorticoids might improve survival.

Previous meta-analyses assessing the effects of systemic glucocorticoids on the mortality of COVID-19 had different designs and showed conflicting results due to substantial heterogeneity in outcomes, the inclusion of both observational studies and randomised trials, the statistical methodology and the inclusion of patients without a confirmed diagnosis of COVID-19 in some reports (eTable5 in the Supplement) [5], [33], [37], [38], [39], [40], [41], [42], [43], [44], [45], [46], [47], [48], [49], [50], [51], [52], [53]. Interestingly, only one other meta-analysis [5] used the Hartung and Knapp adjustment [54] to account for the uncertain heterogeneity amongst trials. Had this test not been applied in our analysis, glucocorticoids would have been associated with a 13% (p = 0.03) reduction in-hospital all-cause mortality in the overall population. Despite some differences in potency and activity [55], our findings suggest no difference in efficacy amongst glucocorticoids. Two recent trials found that inhaled budesonide improved time to recovery in ambulatory patients with a mild COVID-19 infection [56], [57], indicating that there might still be a role for inhaled steroids in the management of milder COVID-19 infections.

We expand previous observations not only by including a greater number of randomised trials, but also reporting that glucocorticoids reduced risk of worsening renal function compared to standard care or placebo by 37%. This could be due to beneficial effects of glucocorticoids on renal endothelial inflammation. Alternatively, by increasing salt and water reabsorption, off-target mineralocorticoid effects of glucocorticoids, plasma volume and systolic blood pressure may increase, thereby maintaining glomerular perfusion and filtration rate.

The rate of reported cardiovascular complications in COVID-19 trials was lower than expected based on observational studies [6] which may have several explanations. For instance, the RECOVERY trial, which contributed 80% of patients in this meta-analysis, was a UK national effort to provide a rapid response on the effects of several treatments, including dexamethasone, on mortality of patients with a severe COVID-19 infection; only essential data were collected. Busy research staff, absence of monitoring, and difficulties in diagnosing cardiovascular events such as heart failure in patients who were severely breathless or ventilated might have also contributed to underreporting.

There are several limitations to our meta-analysis. The great majority of patients included in this meta-analysis are from the RECOVERY trial. Many trials, such as CAPE COVID, CoDEX and REMAP-CAP, were stopped upon the release of the RECOVERY findings because it was no longer considered ethical to withhold steroids in sicker patients. Other trials have not been published (ie: DEXA-COVID19 (NCT04325061) [58] and Steroids-SARI (NCT04244591)). However, these trials probably enrolled few patients, and their inclusion is unlikely to have affected our results substantially. Moderate heterogeneity and the inclusion of one randomised trial with a high risk of bias weakens the certainty of our findings; the low rate of reported cardiovascular events reduces precision and precludes their further assessment. Due to heterogeneity in patient characteristics, follow-up duration across trials and on type, dose and timing of administration of glucocorticoids, we decided to conduct post-hoc subgroup analysis to investigate the impact of disease severity at randomisation and treatment effect interactions. A recent randomised trial found no statistically significant difference in outcome with 6 mg/day versus 12 mg/day of dexamethasone in 1000 patients with COVID-19 requiring respiratory support, although the trend (adjusted relative risk 0.87 [99% CI, 0.70–1.07]) favoured the higher dose [18, 59]. We did not perform a meta-analysis for other important endpoints, for instance length of hospital stay or intensive care, or post-discharge mortality, as this information was rarely reported. In addition, we did not correct results for demographics, such as age and sex, or comorbidities likely to influence prognosis of patients with COVID-19.

## 5 Conclusions

This systematic review and meta-analysis of randomised trials of patients admitted to hospital with COVID-19 suggests that administration of systemic glucocorticoids might reduce mortality for those requiring respiratory support but increase mortality for those who do not. Premature administration of steroids may be harmful in this setting. However, further trials are required to refine the decision on whether to administer or withhold steroids for patients in whom the need for respiratory support is itself uncertain (eg: hypoxaemia that is not yet considered severe enough to require oxygen or patients who respond well to oxygen and do not require ventilator support). Uncertainty also persists about the optimal dose and length of treatment in this very heterogeneous population.

## CRediT authorship contribution statement

EC, AOMR, PP and PM designed the study and drafted the manuscript; JGFC and TG provided supervision; EC and AOMR conducted the statistical analysis, supervised by DB. All authors critically revised and approved the final version of the manuscript.

## Funding Sources

Our work was supported by the British Heart Foundation (BHF) [Grant PG/19/84/34771, PG/21/10541, PG/21/10634]; the European Research Council [Project Identiﬁer: 726318]; and the Italian Ministry of University and Research (MIUR) PRIN 2017 [2017NKB2N4_003]. EC is supported by a European Society of Cardiology Research Grant.

## Conflict of Interest

The authors declare that the research was conducted in the absence of any commercial or financial relationships that could be construed as a potential conflict of interest.
