# Supplementary material for: Systemic administration of glucocorticoids, cardiovascular complications and mortality in patients hospitalised with COVID-19, SARS, MERS or influenza: A systematic review and meta-analysis of randomised trials
Source: Pharmacol Res. 2022 Feb;176:106053. doi: 10.1016/j.phrs.2021.106053 (PMC8719379; doi:10.1016/j.phrs.2021.106053)
Supplement: Supplementary file 1 — Supplementary material. [file mmc1.docx]

**Supplementary Online Content**

**Search Strategy**

**eTable1.** Risk of Bias Assessment for In-Hospital Mortality using Cochrane RoB2 Tool.

**eTable2.** Risk of Bias Assessment for Worsening Renal Function using Cochrane RoB2 Tool.

**eTable3.** In-Hospital Mortality and Cardio-Renal Events in 11 Randomised Trials.

**eTable4.** Measured vs. Expected Incidence of Cardiovascular, Metabolic and Renal Outcomes.

**eTable5.** Published Meta-Analyses Assessing the Use of Systemic Glucocorticoids in COVID-19 Patients

**eFigure1.** Funnel Plot of the 11 Randomised Trials Included in the Meta-Analysis.

**eFigure2.** Sensitivity Analysis Excluding Randomised Trials at High Risk of Bias for Mortality Outcomes.

**eFigure3.** Subgroup Analysis of In-Hospital Mortality by Type of Glucocorticoid Used.

**eFigure4.** Subgroup Analysis for Effect of Standard Care/Tocilizumab vs. Placebo on Mortality Outcomes.

**eFigure5.** Cardiovascular Effects of Glucocorticoids in Hospitalized COVID-19 Patients.

**References**

**Search Strategy**

**Condition-related terms:**

COVID-19 OR COVID19 OR COVID-19 Virus OR COVID-19 Viruses OR COVID19 Virus OR Coronavirus Disease 2019 Virus OR COVID-19 Virus Infection OR COVID-19 Virus Disease OR Coronavirus 2 OR Coronaviruses OR Coronavirus OR Coronavirus Disease 19 OR Coronavirus Disease-19 OR Coronavirus Pneumonia OR 2019-nCoV OR 2019nCoV OR 2019 Novel Coronavirus OR 2019 Novel Coronaviruses OR 2019 Novel Coronavirus Disease OR 2019 Novel Coronavirus Infection OR 2019-nCoV Disease OR Novel Coronavirus 2019 OR SARS-CoV-2 OR SARS-CoV-2 Virus OR SARS-CoV-2 Viruses OR SARS OR SARS Virus OR SARS Coronavirus 2 OR SARS CoV 2 Virus OR SARS-Associated Coronavirus OR SARS-Related Coronavirus OR SARS-CoV OR SARS-nCoV OR SARS CoV 2 Infection OR SARS-CoV-2 Infection OR SARS Coronavirus 2 Infection OR Severe Acute Respiratory Syndrome Coronavirus 2 OR Severe Acute Respiratory Syndrome OR Severe Acute Respiratory Syndrome-Related Coronavirus OR Severe Acute Respiratory Syndrome Related Coronavirus OR Severe Acute Respiratory Syndrome Coronavirus OR Severe Acute Respiratory Syndrome Virus. Coronavirus 1 OR Coronaviruses OR Coronavirus OR SARS-CoV-1 OR SARS OR SARS Virus OR SARS Coronavirus 1 OR SARS-Associated Coronavirus OR SARS-Related Coronavirus OR SARS-CoV OR Severe Acute Respiratory Syndrome Coronavirus 1 OR Severe Acute Respiratory Syndrome OR Severe Acute Respiratory Syndrome-Related Coronavirus OR Severe Acute Respiratory Syndrome Related Coronavirus OR Severe Acute Respiratory Syndrome Coronavirus OR Severe Acute Respiratory Syndrome Virus. Middle East Respiratory Syndrome OR Middle East Respiratory Syndrome Coronavirus OR Middle East Respiratory Syndrome-Related Coronavirus OR Middle East Respiratory Syndrome Related Coronavirus OR MERS OR MERS Viruses OR MERS Virus OR MERS-CoV. Influenza OR Influenzas OR Human Flu OR Flu OR Influenza A OR Influenza A Virus OR Influenza Viruses Type A OR Influenza B OR Influenza B Virus OR Influenza B viruses OR Influenza Viruses Type B OR Influenza C OR Influenza C Virus OR Influenza C Viruses OR Influenza Viruses Type C OR Influenza Pneumonia OR Viral Pneumonia OR Viral Pneumonias.

**Intervention-related terms:**

Dexamethasone OR Dexametasone OR Methylprednisolone OR Methylprednisone OR Hydrocortisone OR Prednisone OR Prednisolone OR Corticosteroids OR Corticosteroid OR Glucocorticosteroids OR Glucocorticosteroid OR Glucocorticoids OR Glucocorticoid.

**Study Design-related terms:**

Randomised Controlled Trial OR Randomized Controlled Trial OR Randomised Controlled Trials OR Randomized Controlled Trials OR Randomised Clinical Trial OR Randomized Clinical Trial OR Randomised Clinical Trials OR Randomized Clinical Trials OR Controlled Trial OR Controlled Study OR Trial OR Randomisation OR Randomisation OR Controlled Clinical Trial OR Clinical Trial.

**eTable1. Risk of Bias Assessment for In-Hospital Mortality using Cochrane RoB2 Tool.**

| **Trial** | **Randomisation Process** | **Deviations from the Intended Interventions** | **Missing Outcome Data** | **Measurement of the Outcome** | **Selection of the Reported Result** | **Overall** |
| --- | --- | --- | --- | --- | --- | --- |
| **Angus**  **2020** | Low | Low | Low | Low | Low | **Low** |
| **Corral-Gudino**  **2021** | Low | Low | Low | Low | Some concerns | **Some concerns** |
|  | No pre-specified analyses in sufficient detail (No protocol/statistical analysis plan published prior to main report). | | | | | |
| **Dequin**  **2020** | Low | Low | Low | Low | Low | **Low** |
| **Edalatifard**  **2020** | Low | Some  concerns | Low | Low | Some concer/ns | **Some concerns** |
|  | Open label design. Results reported are of per-protocol analysis; 6 patients were excluded from the standard care group because they received glucocorticoids. Authors mention that the ITT analysis outcomes did not differ significantly from per-protocol analysis although results were not reported. No prespecified analysis in sufficient detail (no protocol/statistical analysis plan published prior to main report). There is no information on the time point for assessment of in-hospital mortality. | | | | | |
| **Horby**  **2021** | Low | Low | Low | Low | Low | **Low** |
| **Jamaati**  **2021** | Some  concerns | Low | Low | Low | Some concerns | **Some concerns** |
|  | No information on allocation concealment. No prespecified analysis in sufficient detail (no protocol/statistical analysis plan published prior to main report). | | | | | |
| **Jeronimo**  **2021** | Low | Low | Low | Low | Low | **Low** |
| **Munch**  **2021** | Low | Low | Low | Low | Low | **Low** |
| **Rashad**  **2021** | Some  concerns | High | High | Low | Some concerns | **High** |
|  | No information on allocation concealment. Open label trial, 40 patients (~27%) were excluded from primary analysis after randomisation due to early mortality. No prespecified analysis in sufficient detail (no protocol/statistical analysis plan published prior to main report). | | | | | |
| **Tang**  **2021** | Low | Low | Low | Low | Some concerns | **Some concerns** |
|  | No prespecified analysis in sufficient detail (no protocol/statistical analysis plan published prior to main report). | | | | | |
| **Tomazini**  **2020** | Low | Some  concerns | Low | Low | Low | **Some concerns** |
|  | Open label design. | | | | | |

**eTable2. Risk of Bias Assessment for Worsening Renal Function using Cochrane RoB2 Tool.**

| **Trial** | **Randomisation Process** | **Deviations from the Intended Interventions** | **Missing Outcome Data** | **Measurement of the Outcome** | **Selection of the Reported Result** | **Overall** |
| --- | --- | --- | --- | --- | --- | --- |
| **Horby**  **2021** | Low | Low | Low | Low | Low | **Low** |
| **Jeronimo**  **2021** | Low | Low | Low | Low | Low | **Low** |
| **Rashad**  **2021** | Some  concerns | High | High | Low | Some concerns | **High** |
|  | No information on allocation concealment. Open label trial and 40 patients (~27%) were excluded from primary analysis after randomisation due to early mortality. No prespecified analysis in sufficient detail (no protocol/statistical analysis plan published prior to main report). No explanation of method of outcome assessment. | | | | | |
| **Tomazini**  **2020** | Low | Some  concerns | Low | Low | Low | **Some concerns** |
|  | Open label design. | | | | | |

**eTable3. In-Hospital Mortality and Cardio-Renal Events in 11 Randomised Trials.**

| **Trial** | **Group** | **Events n/N (%)** | | | | | | | | | |
| --- | --- | --- | --- | --- | --- | --- | --- | --- | --- | --- | --- |
|  |  | **14 Days Mortality** | **21-28 Days Mortality** | **Hyperglycaemia**  **Need for Insulin** | **Worsening Renal Function** | **Circulatory Failure** | **Cardiac Arrest**  **Ventricular Arrhythmia** | **Other Arrhythmias** | **MI** | **DVT**  **PE** | **Stroke** |
| **Angus**  **2020** | **Total** | 63/379  (17) | 111/379  (29) | - | - | - | - | - | - | - | - |
|  | **Hydrocortisone** | 47/278  (17) | 78/278  (28) | - | - | - | - | - | - | - | - |
|  | **Control** | 16/101  (16) | 33/101  (33) | - | - | - | - | - | - | - | - |
| **Definitions:** 14-days mortality (WHO scale (range, 0-8, 8 = death), 21-days Mortality (Sub-component of organ support-free days, death = -1). | | | | | | | | | | | |
| **Corral-Gudino 2021** | **Total** | - | 12/64  (19) | 9/64  (14) | - | - | 0/64  (0) | 0/64  (0) | - | - | - |
|  | **Methylprednisolone** | - | 7/35  (20) | 9/35  (26) | - | - | 0/35  (0) | 0/35  (0) | - | - | - |
|  | **Control** | - | 5/29  (17) | 0/29  (0) | - | - | 0/29  (0) | 0/29  (0) | - | - | - |
| **Definitions:** 28-days in-hospital all-cause mortality (Component of a composite outcome). Hyperglycaemia (>180 mg/dl based on the fasting blood glucose during first 6 days of inclusion, MP duration). Unplanned (Clinically significant arrhythmias). | | | | | | | | | | | |
| **Dequin**  **2020** | **Total** | - | 31/149  (21) | - | - | - | 1/149  (1) | - | - | 1/149  (1) | - |
|  | **Hydrocortisone** | - | 11/76  (14) | - | - | - | 1/76  (1) | - | - | 1/76  (1) | - |
|  | **Control** | - | 20/73  (27) | - | - | - | 0/73  (0) | - | - | 0/73  (0) | - |
| **Definitions:** 21-days mortality (Component of status on day 21 5-item scale). Episode of cardiac arrest due to pulmonary embolism. | | | | | | | | | | | |
| **Edalatifard 2020** | **Total** | - | 14/62  (23) | - | - | 3/62  (5) | - | - | - | - | - |
|  | **Methylprednisolone** | - | 2/34  (6) | - | - | 1/34  (3) | - | - | - | - | - |
|  | **Control** | - | 12/28  (43) | - | - | 2/28  (7) | - | - | - | - | - |
| **Definitions:** 21-days mortality (21-days rather than 15-days classification estimated from KM curve and average time-to-event). Oedema and shock. | | | | | | | | | | | |
| **Horby**  **2021** | **Total** | - | 1592/6425  (25) | 2/6425  (0) | 403/6228  (6) | - | 20/2913  (1) | 157/2913  (5) | - | - | 9/6425  (0) |
|  | **Dexamethasone** | - | 482/2104  (23) | 2/2104  (0) | 89/2034  (4) | - | 8/973  (1) | 46/973  (5) | - | - | 4/2104  (0) |
|  | **Control** | - | 1110/4321  (26) | 0/4321  (0) | 314/4194  (8) | - | 12/1940  (1) | 111/1940  (6) | - | - | 5/4321  (0) |
| **Definitions:** 28-days mortality. Hyperglycaemia. Renal replacement therapy (renal dialysis or hemofiltration). Ventricular tachycardia or fibrillation. Atrial flutter or atrial fibrillation, other supraventricular tachycardia, and atrioventricular block requiring intervention. Stroke. Values represent number of events/total number (%). | | | | | | | | | | | |
| **Jamaati 2021** | **Total** | - | 31/50  (62) | - | - | - | - | - | - | - | - |
|  | **Dexamethasone** | - | 16/25  (64) | - | - | - | - | - | - | - | - |
|  | **Control** | - | 15/25  (60) | - | - | - | - | - | - | - | - |
| **Definitions:** 28-days mortality. | | | | | | | | | | | |
| **Jeronimo 2020** | **Total** | 127/416  (31) | 159/416  (38) | 190/351  (54) | 62/385  (16) | 31/393  (8) | - | - | - | - | - |
|  | **Methylprednisolone** | 60/209  (29) | 79/209  (38) | 104/177  (59) | 29/191  (15) | 17/194  (9) | - | - | - | - | - |
|  | **Control** | 67/207  (32) | 80/207  (39) | 86/174  (49) | 33/194  (17) | 14/199  (7) | - | - | - | - | - |
| **Definitions:** 14-days mortality. 28-days mortality. Need for insulin therapy until day 28. Need for dialysis until day 28. Shock under treatment of norepinephrine. | | | | | | | | | | | |
| **Munch 2021** | **Total** | - | 8/30  (27) | - | - | - | - | - | - | - | - |
|  | **Hydrocortisone** | - | 6/16  (38) | - | - | - | - | - | - |  | - |
|  | **Control** | - | 2/14  (14) | - | - | - | - | - | - |  | - |
| **Definitions:** All-cause 28-days mortality. | | | | | | | | | | | |
| **Rashad**  **2021** | **Total** | 105/149  (70) | - | 15/109  (14) | 5/109  (5) | - | - | - | - | 4/109  (4) | - |
|  | **Dexamethasone** | 45/75  (60) | - | 9/63  (14) | 2/63  (3) | - | - | - | - | 1/63  (2) | - |
|  | **Control** | 60/74  (81) | - | 6/46  (13) | 3/46  (7) | - | - | - | - | 3/46  (7) | - |
| **Definitions:** 14-days mortality. Hyperglycaemia. Renal impairment. Pulmonary embolism. | | | | | | | | | | | |
| **Tang**  **2021** | **Total** | 1/86  (1) | - | 10/86  (12) | - | - | - | - | - | - | - |
|  | **Methylprednisolone** | 0/43  (0) | - | 3/43  (7) | - | - | - | - | - | - | - |
|  | **Control** | 1/43  (2) | - | 7/43  (16) | - | - | - | - | - | - | - |
| **Definitions:** 14-days mortality. Hyperglycaemia with need for additional therapy. | | | | | | | | | | | |
| **Tomazini 2020** | **Total** | 119/299  (40) | 176/299  (59) | 91/299  (30) | 1/299  (0) | 1/299  (0) | - | - | 3/299  (1) | 3/299  (1) | - |
|  | **Dexamethasone** | 54/151  (36) | 85/151  (56) | 48/151  (32) | 0/151  (0) | 0/151  (0) | - | - | 1/151 (1) | 1/151 (1) | - |
|  | **Control** | 65/148  (44) | 91/148  (61) | 43/148  (29) | 1/148  (1) | 1/148  (1) | - | - | 2/148 (1) | 2/148 (1) | - |
| **Definitions:** 15-day mortality. (6-point ordinal scale at day 15, 6 = death). All-cause 28-days mortality. Insulin use for hyperglycaemia or unspecified hyperglycaemia assessed daily until day 14. Nephropathy in transplanted kidney. Cardiogenic shock. Acute MI. Deep vein thrombosis and pulmonary embolism. Values represent number of events/total number (%). | | | | | | | | | | | |
| MI: myocardial infarction, DVT: deep vein thrombosis, PE: pulmonary embolism, MP: methylprednisolone. | | | | | | | | | | | |

**eTable4. Measured vs. Expected Incidence of Cardiovascular, Metabolic and Renal Outcomes.**

| **Event** | **Patients in this Meta-Analysis** | **Reported Number of Events** | **Measured Incidence** | **Expected Number of Events** | **Expected Incidence^a^** |
| --- | --- | --- | --- | --- | --- |
| **Worsening Renal Function** | **8,109** | **471** | **5.8%** | **414** | **5.1%** |
| **Hyperglycaemia or Need for Insulin Therapy** |  | **317** | **3.9%** | **2,141** | **26.4%** |
| **Circulatory Failure** |  | **232** | **2.9%** | **2,011** | **24.8%** |
| **Cardiac Arrest or Ventricular Arrhythmia** |  | **21** | **0.3%** | **219** | **2.7%** |
| **Other Arrhythmias** |  | **157** | **1.9%** | **795** | **9.8%** |
| **MI** |  | **3** | **0.04%** | **138** | **1.7%** |
| **DVT/PE** |  | **8** | **0.1%** | **843** | **10.4%** |
| **Stroke** |  | **9** | **0.1%** | **97** | **1.2%** |
| ^a^Expected incidence is derived from^1^ for all outcomes except hyperglycaemia, which was estimated following a pooled mean approach from 14,302 patients across 7 studies ^2-8^. MI: myocardial infarction, DVT: deep vein thrombosis, PE: pulmonary embolism. | | | | | |

**eTable5. Published Meta-Analyses Assessing the Use of Systemic Glucocorticoids in COVID-19 Patients**

| **Reference**  ***First Author*** | **Search Date Limit** | **Included Patients** | **Included Studies (n)** | **Mortality Effect**  **Estimate** | **Notes** | **Hartung-Knapp**  **Adjustment** |
| --- | --- | --- | --- | --- | --- | --- |
| ^9^  *Cano EJ* | Jul. 22, 2020 | 16,336 | Observational (31)  **Randomised Trials** **(1)**  Horby | OR^a^: 2.30  (1.45-3.63, p= 0.0004)  OR^b^: 0.65  (0.51-0.83, p= 0.0006) | ^a^Overall, I^2^ = 90%, REM.  ^b^Severely ill patients (ARDS, mechanically ventilated, or critically ill). 8 studies (1,924 patients). I^2^ = 29%, FEM. | No |
| ^10^  *Chaudhuri D* | Sep. 6, 2020 | 2,826 | Observational (0)  **Randomised Trials (8)**  Tomazini, Angus, Dequin, Horby, Jeronimo, DEXA-COVID19, Munch, Steroids-SARI. | RR^a^: 0.82  (0.72-0.95, p=0.007) | ^a^COVID-19 and nonCOVID-19 ARDS. 16 trials (2,740 patients). I^2^ = 46%, REM. | No |
| ^11^  *Hasan SS* | Apr. 13, 2021 | 652 | Observational (0)  **Randomised Trials (5)**  Steroids-SARI, Tang, Jeronimo, Corral-Gudino, Edalatifard. | OR: 0.64  (0.29-1.43, p=0.06) | I^2^ = 55%, REM. | No |
| ^12^  *Li H* | Mar. 20, 2020 | 5,249 | Observational (10)  **Randomised Trials (1),** *non-COVID* | RR^a^: 1.07  (0.81-1.42, p=0.68) | ^a^8 observational studies (COVID-19 *n*= 327; SARS n= 3,273; MERS n = 309). I^2^ = 80%, REM. | No |
| ^13^  *Li J* | Oct. 7, 2020 | 45,935 | Observational (71)  **Randomised Trials (10)**  Corral-Gudino, Horby, Jeronimo, Tomazini, Angus, Edalatifard, Dequin, Munch, DEXA-COVID19, Steroids-SARI. | RR^a^: 0.88  (0.82-0.94, p<0.001) | ^a^COVID-19 patients. 10 trials, I^2^ = 26%, REM. | No |
| ^14^  *Lu S* | Mar. 31, 2020 | 13,815 | Observational (22)  **Randomised Trials (1),** *non-COVID* | RR^a^: 2.00  (0.69- 5.75, p=0.000) | ^a^4 observational COVID-19 studies.  I^2^ = 91%, REM. | No |
| ^15^  *Ma S* | Oct. 1, 2020 | 6,250 | Observational (0)  **Randomised Trials (7)**  Angus, Corral-Gudino, Dequin, Edalatifard, Horby, Jeronimo, Tomazini. | RR: 0.85  (0.73-0.99, p=0.04) | I^2^ = 43%, REM. | No |
| ^16^  *Pasin L* | Nov. 2020* | 7,692 | Observational (0)  **Randomised Trials (5)**  Angus, Dequin, Horby, Jeronimo, Tomazini. | RR^a^: 0.89  (0.82-0.96, p=0.003)  RR^b^: 0.85  (0.72-1.00, p=0.05)  RR^c^: 1.23  (1.00-1.62, p=0.05) | *Publication date.  ^a^Overall, I^2^ = 0%.  ^b^Mechanically ventilated, I^2^ = 58%.  ^c^Not requiring oxygen, I^2^ = 0%. | No |
| ^17^  *Pulakurthi YS* | Mar. 10, 2021 | 7,737 | Observational (0)  **Randomised Trials (8)**  Angus, Corral-Gudino, Dequin, Edalatifard, Horby, Jeronimo, Tang, Tomazini. | OR: 0.85  (0.76-0.95, p=0.003) | I^2^ = 40%, REM. | No |
| ^18^  *Sahilu T* | Jan. 30, 2021 | 14,659 | Observational (27)  **Randomised Trials (5)**  Angus, Dequin, Horby, Jeronimo, Tomazini. | RR^a^: 0.95  (0.80-1.13, p=0.57)  RR^b^: 0.89  (0.62–1.27, p=0.52)  RR^c^: 1.10  (1.03–1.19, p=0.007) | ^a^26 studies (13,565 patients). I^2^ = 78%, REM.  ^b^5 studies (1,564 critically ill patients). I^2^ = 78%, REM.  ^c^23 studies reporting disease severity. I^2^ = 99%, REM. | No |
| ^19^  *Sarkar S* | Aug. 19, 2020 | 15,754 | Observational (10)  **Randomised Trials (2)**  Corral-Gudino & Horby. | OR: 1.94  (1.11-3.40, p=0.02) | I^2^ = 96%, REM. | No |
| ^20^  *Sarma P* | Aug. 8, 2020 | 5,787 | Observational (12)  **Randomised Trials (3)**  Corral-Gudino, Horby, Jeronimo. | RR: 0.83  (0.76-0.91, p<0.0001) | Severe to critical COVID-19 patients. I^2^ = 49%, FEM. | No |
| ^21^  *WHO (REACT) Working Group* | Apr. 6, 2020 | 1,703 | Observational (0)  **Randomised Trials (7)**  Angus, Horby, Tomazini, Dequin, Munch, DEXA-COVID19, Steroids-SARI. | OR^a^: 0.66  (0.53-0.82, p<0.001) OR^b^: 0.70  ( 0.48-1.01, p=0.053) | ^a^FEM analysis. I^2^ = 16%.  ^b^REM analysis. | Yes |
| ^22^  *Tlayjeh H* | Jul. 20, 2020 | 16,977 | Observational (19)  **Randomised Trials (1)**  Horby | RR: 0.91  (0.71-1.16, p<0.01) | 9 observational studies and 1 randomised trial with 10,278 patients. I^2^ = 81%. | No |
| ^23^  *van Paassen J* | Oct. 1, 2020 | 20,197 | Observational (39)  **Randomised Trials (5)**  Angus, Dequin, Horby, Jeronimo, Tomazini.. | OR^a^: 0.72  (0.57-0.87)  OR^b^: 0.84  (0.69-0.99) | ^a^Overall, 22 studies with 14,187 patients. I^2^ = 51%, REM.  ^b^5 randomised trials with 7,645 COVID-19 patients. I^2^ = 31%, REM. | No |
| ^24^  *Wang J* | Jan. 1, 2021 | 15,710 | Observational (44)  **Randomised Trials (7)**  Angus, Horby, Dequin, Jeronimo, Others (Remdesivir Trial, Trial Protocol, Lopinavir-Ritonavir Trial). | OR: 0.35  (0.22-0.56, p<0.00001) | I^2^ = 98%, REM. | No |
| ^25^  *Ye Z* | Apr. 25, 2020 | 20,955 | Observational (31)  **Randomised Trials (21),** *non-COVID* | HR: 2.30  (1.00- 5.29) | Two studies in patients with severe COVID-19. I^2^ = 0%, REM. | No |
| ^26^  *Yousefifard M* | Mar. 2020 | 4,498 | Observational (14)  **Randomised Trials (1),** *non-COVID* | OR: 1.08  (0.34- 3.50) | 5 studies (430 patients) contributed to mortality in COVID-19 population. I^2^ = 79%, REM. | No |
| ^27^  *Cui Y* | Mar. 13, 2021 | 49,057 | Observational (44)  **Randomised Trials (11)**  Angus, Dequin, Edalatifard, Horby, Jamaati, Jeronimo, Tang, Tomazini, Munch, Steroids-SARI, DEXA-COVID19. | OR: 0.91  (0.77–1.07) | Randomised trials (7,893 patients). I^2^ = 63%, REM. | No |

ARDS: acute respiratory distress syndrome. OR: odds ratio. RR: risk ratio. HR: hazards ratio. REM: random effect model. FEM: fixed effect model. REACT: Rapid Evidence Appraisal for COVID-19 Therapies

**eFigure1. Funnel Plot of the 11 Randomised Trials Included in the Meta-Analysis.**

The y-axis shows a transformed measure of standard error (SE) representing study size where low SE associates with larger studies, while x-axis shows risk ratio (RR) representing effect size estimates for the outcome of in-hospital all-cause mortality at the longest follow up. Symmetry of funnel plot indicates low risk of publication bias.


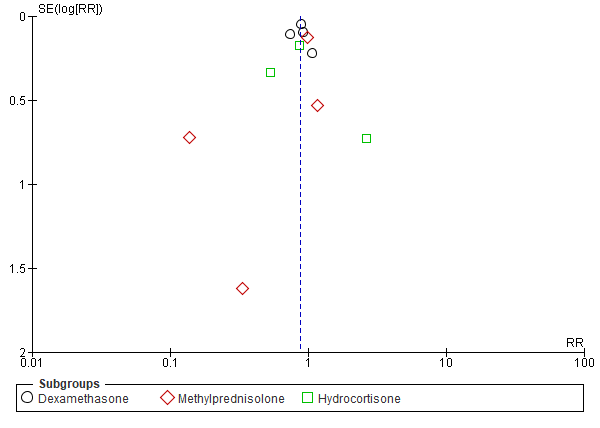


**eFigure2. Sensitivity Analysis Excluding Randomised Trials at High Risk of Bias for Mortality Outcomes.**

Grey-coloured squares show the effect estimate (risk ratios) with the size of each square corresponding to the weight given to each study in the meta-analysis. Horizontal lines represent the 95% CIs corresponding to each effect estimate. The diamond represents the overall effect of intervention with its width representing the overall 95% CI. The I^2^ statistic is a measure of heterogeneity. Risk of bias is reported for each trial assessing five domains: (D1) Randomisation process, (D2) Deviations from the intended interventions, (D3) Missing outcome data, (D4) Measurement of the outcome, (D5) Selection of the reported result.


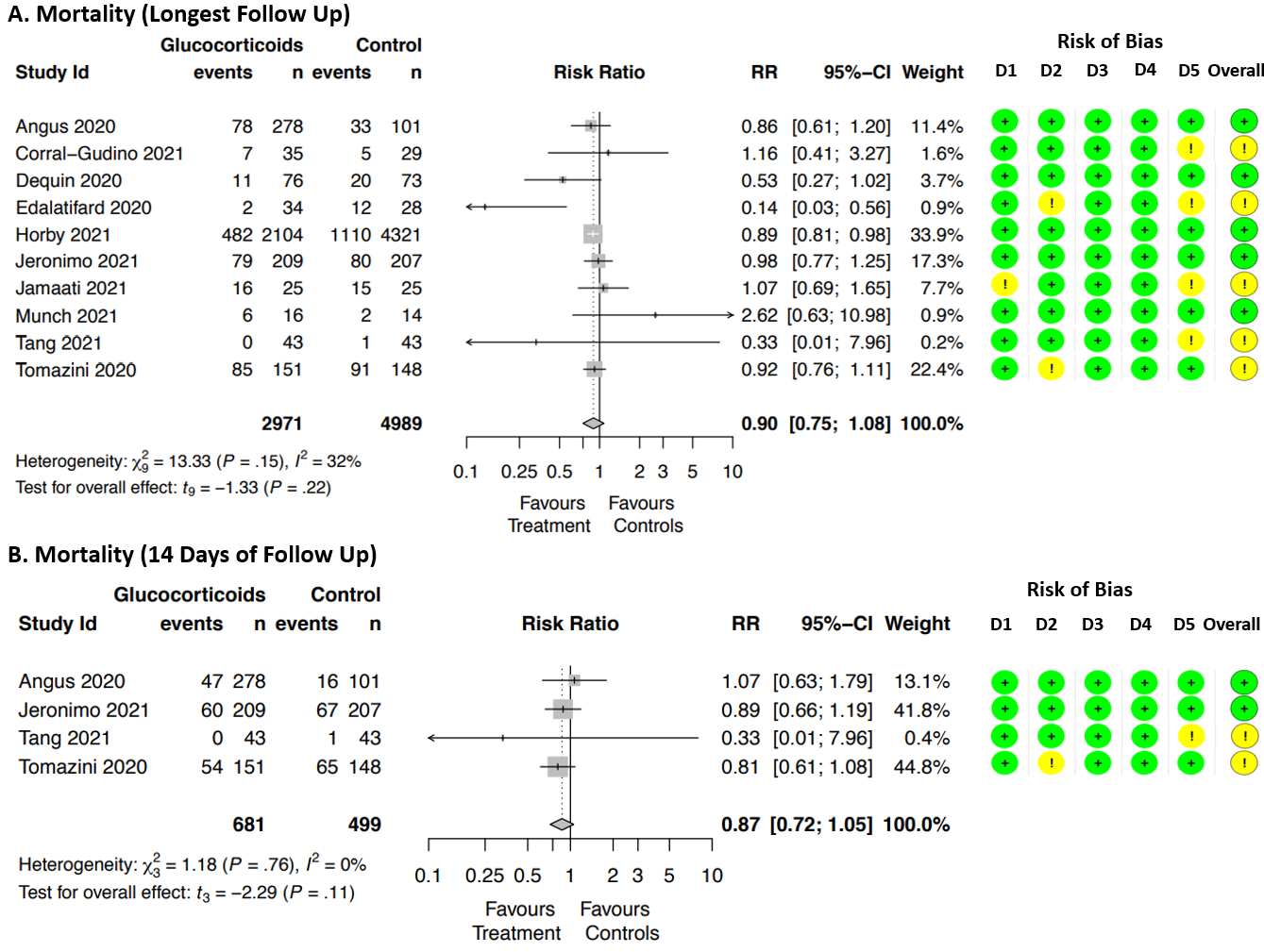


**eFigure3. Subgroup Analysis of In-Hospital Mortality by Type of Glucocorticoid Used.**

Forest plots illustrating the effect of glucocorticoids on the outcome of in-hospital mortality in pre-specified subgroups to detect any effect size modification due to specific treatment allocation. Grey-coloured squares show the effect estimate (risk ratios) with the size of each square corresponding to the weight given to each study in the meta-analysis. Horizontal lines represent the 95% CIs corresponding to each effect estimate. The diamond illustrates the overall effect of intervention with its width representing the overall 95% CI. The I2 statistic represents a measure of heterogeneity. Risk of bias is reported for each trial assessing five domains: (D1) Randomisation process, (D2) Deviations from the intended interventions, (D3) Missing outcome data, (D4) Measurement of the outcome, (D5) Selection of the reported result.


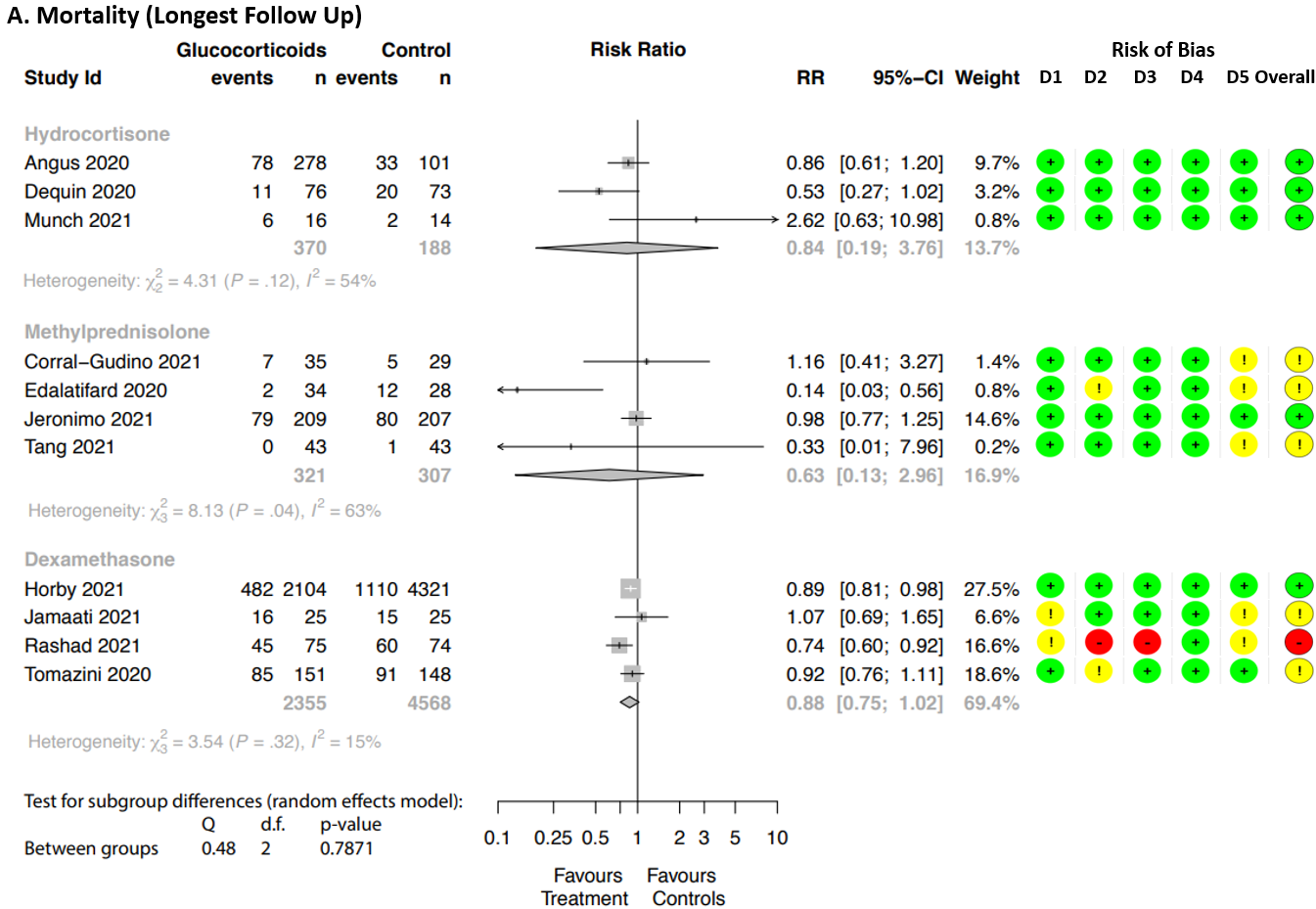


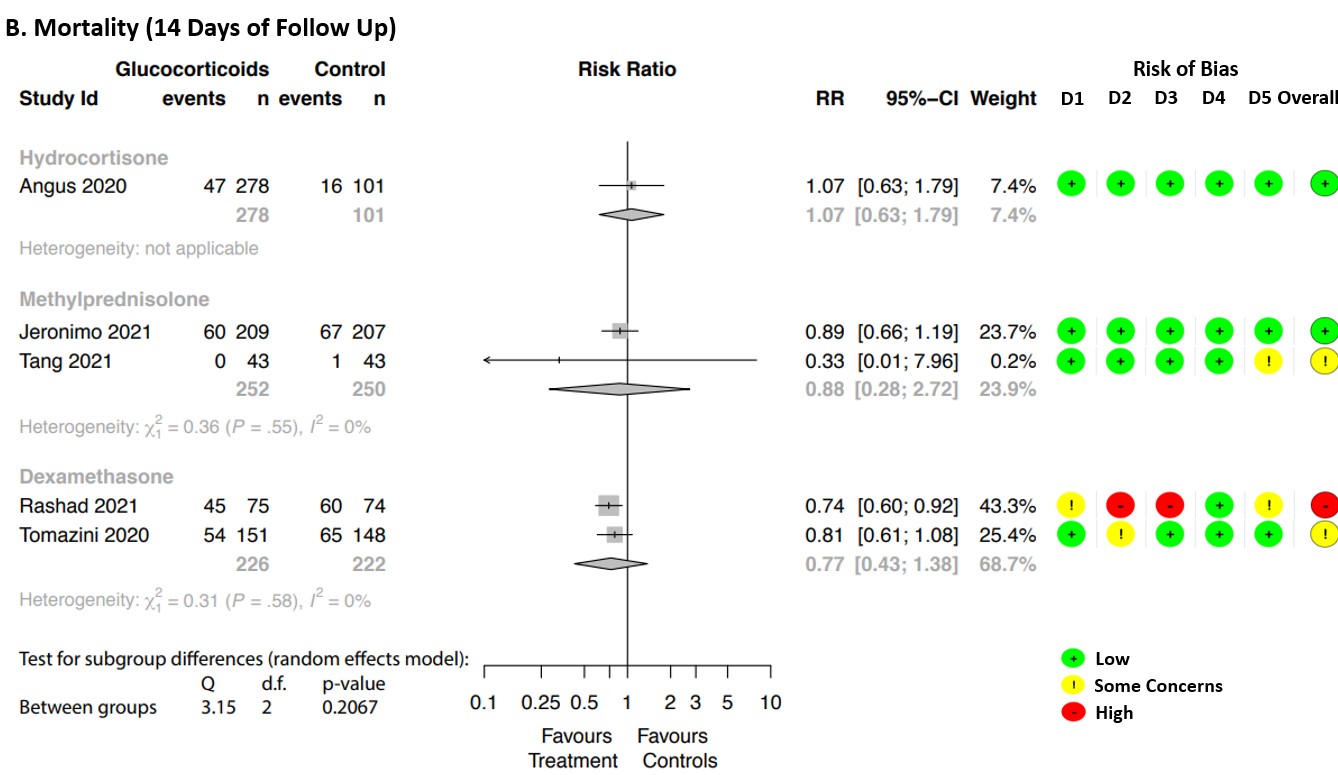


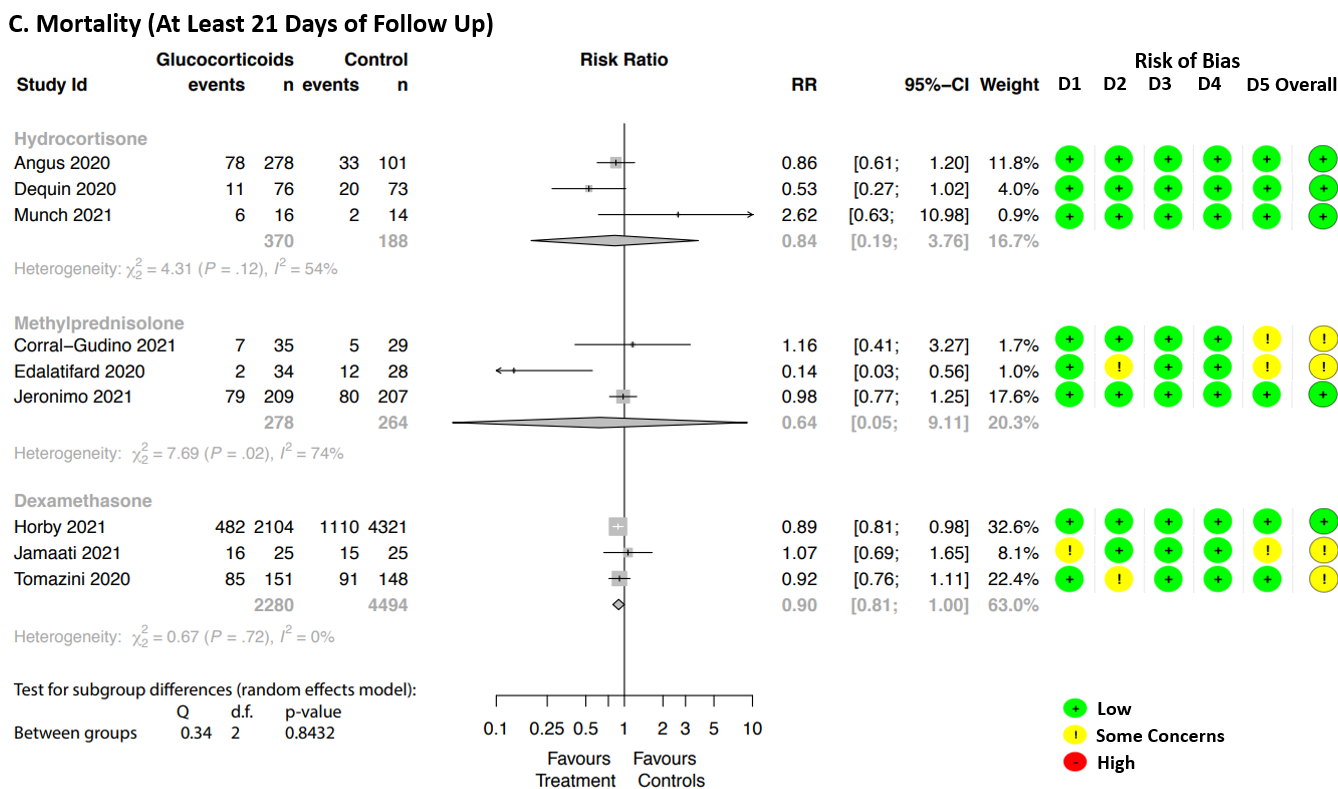


**eFigure 4.** **Subgroup Analysis for Effect of Standard Care/Tocilizumab *vs.* Placebo on Mortality Outcomes.**

Grey-coloured squares show the effect estimate (risk ratios) with the size of each square corresponding to the weight given to each study in the meta-analysis. Horizontal lines represent the 95% CIs corresponding to each effect estimate. The diamond represents the overall effect of intervention with its width representing the overall 95% CI. The I^2^ statistic represents a measure of heterogeneity. Risk of bias is reported for each trial assessing five domains: (D1) Randomisation process, (D2) Deviations from the intended interventions, (D3) Missing outcome data, (D4) Measurement of the outcome, (D5) Selection of the reported result.


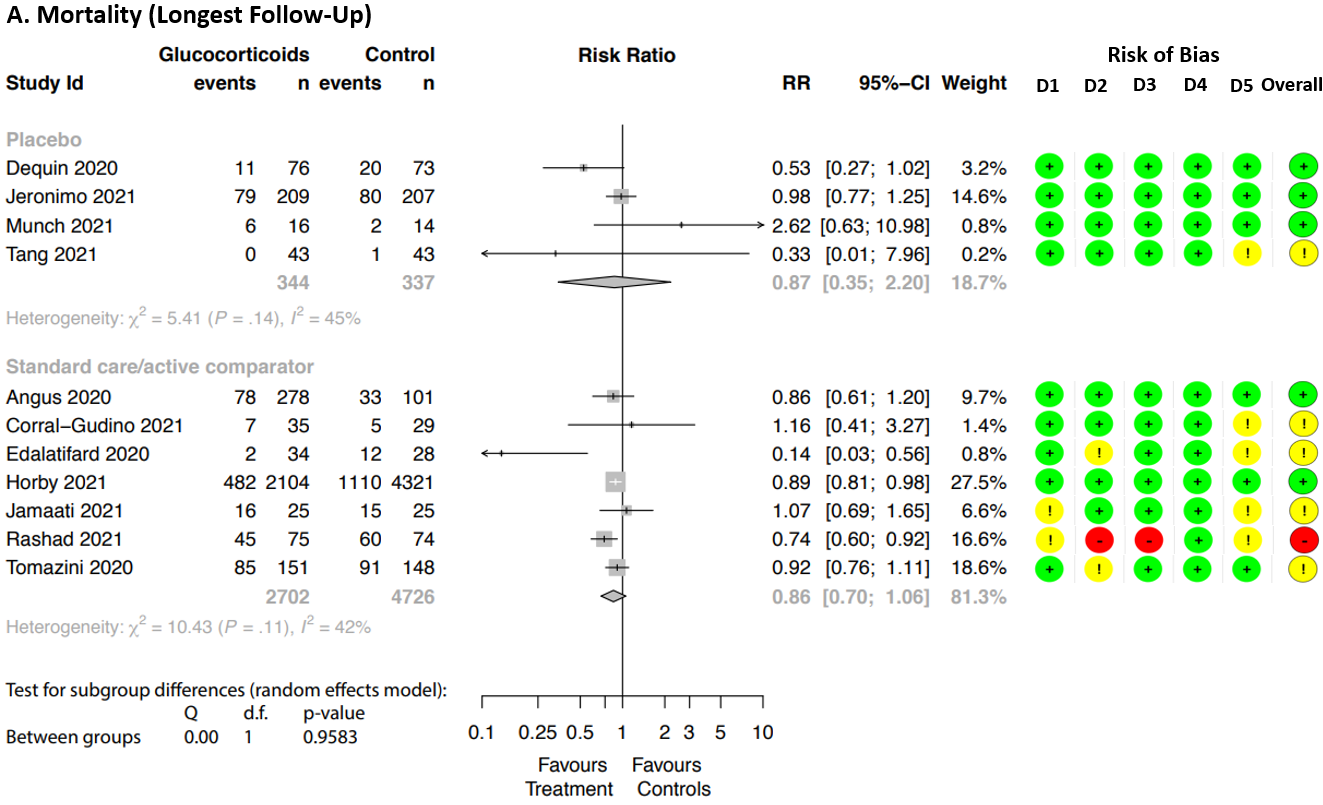


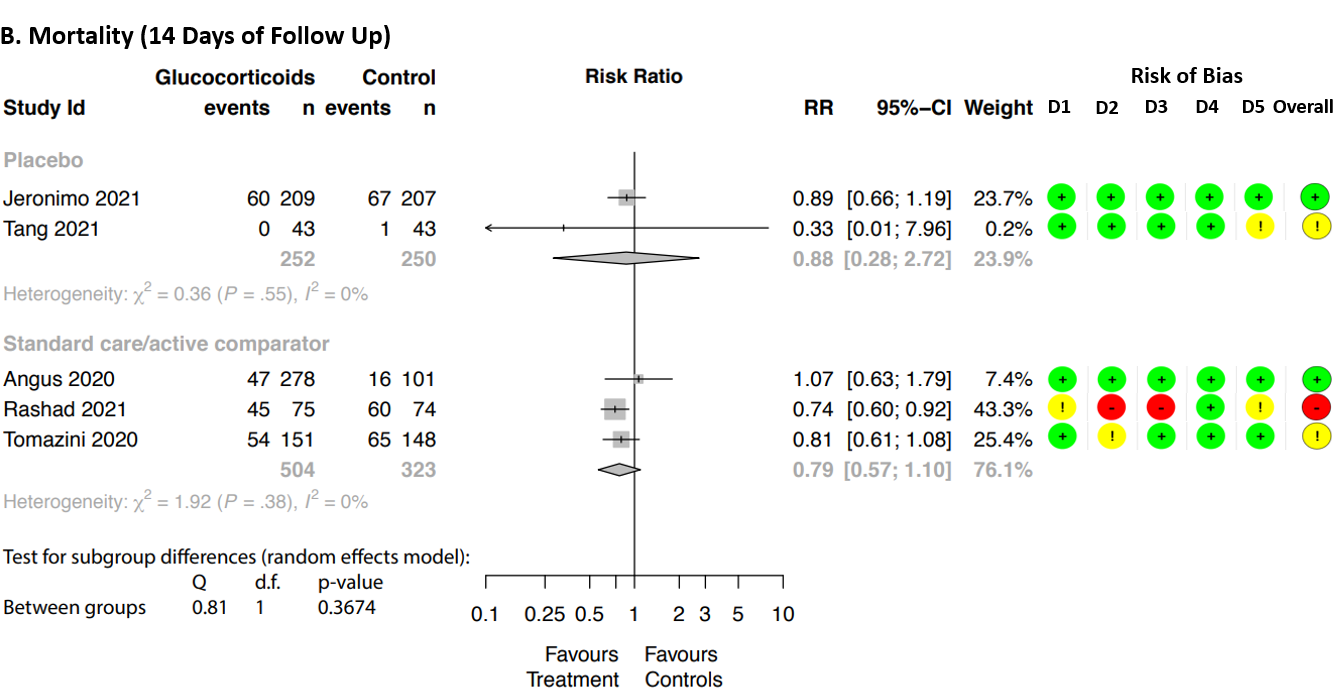

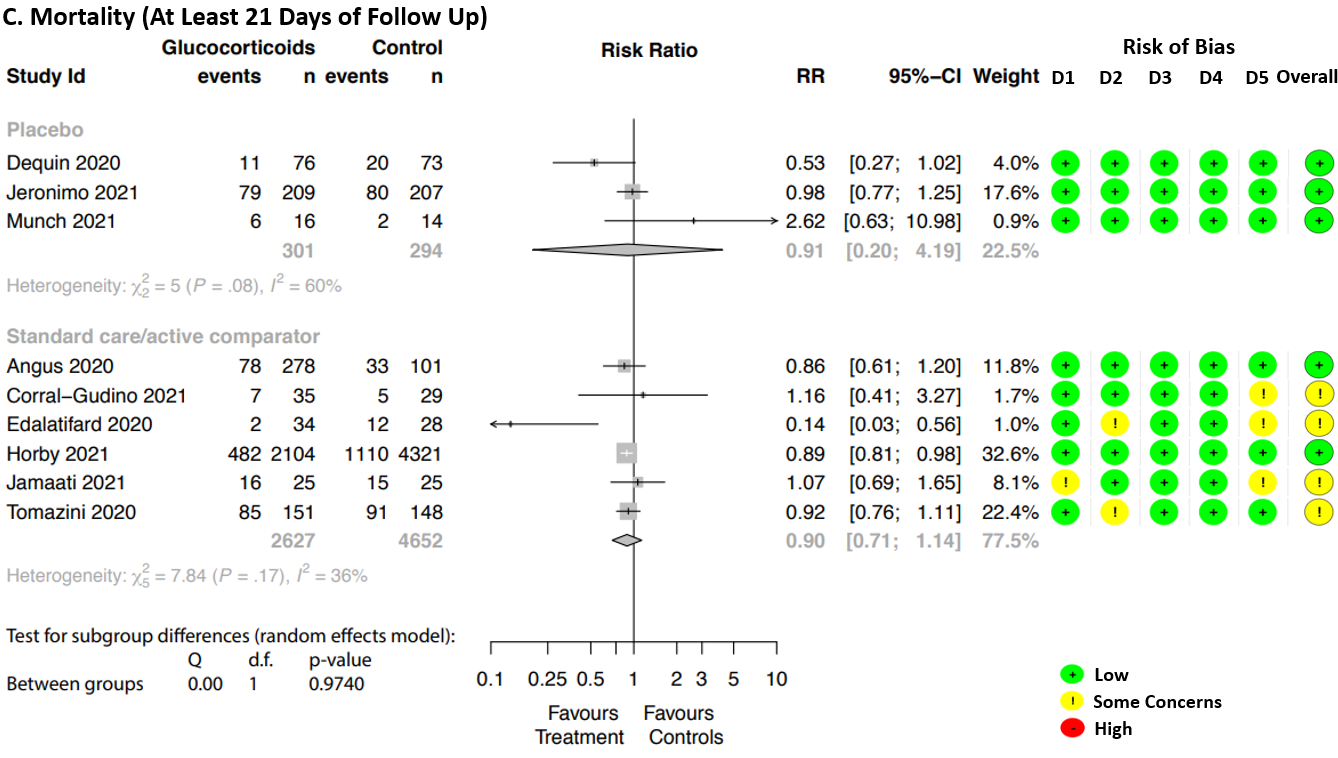


**eFigure5.** **Cardiovascular Effects of Glucocorticoids in Hospitalized COVID-19 Patients**.

Grey-coloured squares show the effect estimate (risk ratios) with the size of each square corresponding to the weight given to each study in the meta-analysis. Horizontal lines represent the 95% CIs corresponding to each effect estimate. The diamond represents the overall effect of intervention with its width representing the overall 95% CI. The I^2^ statistic represents a measure of heterogeneity.


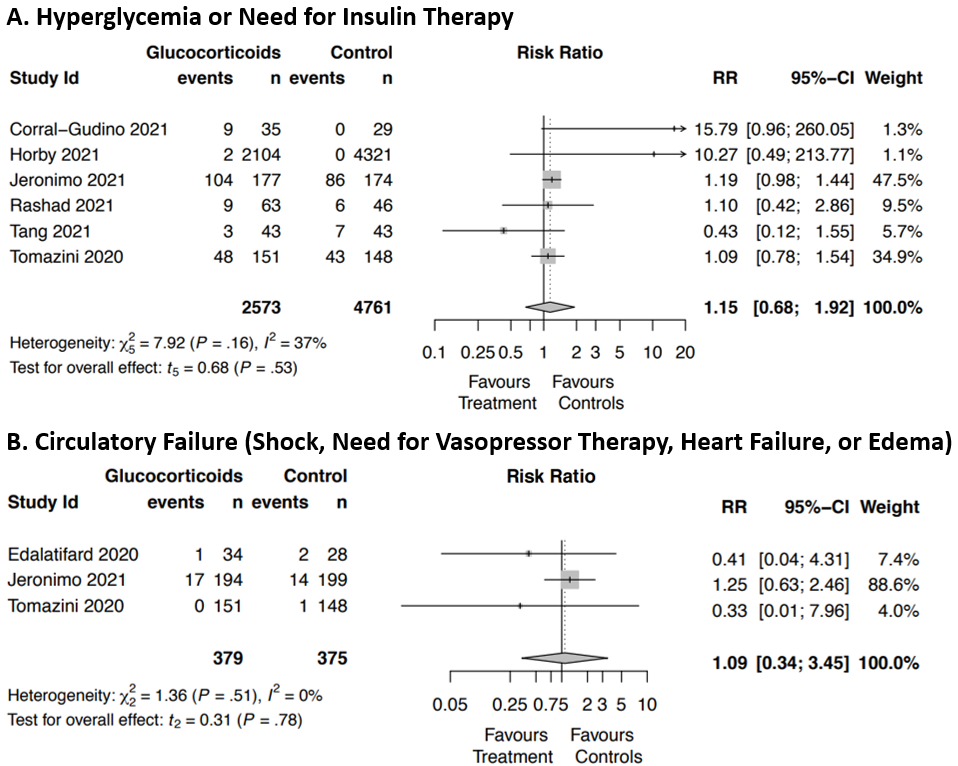


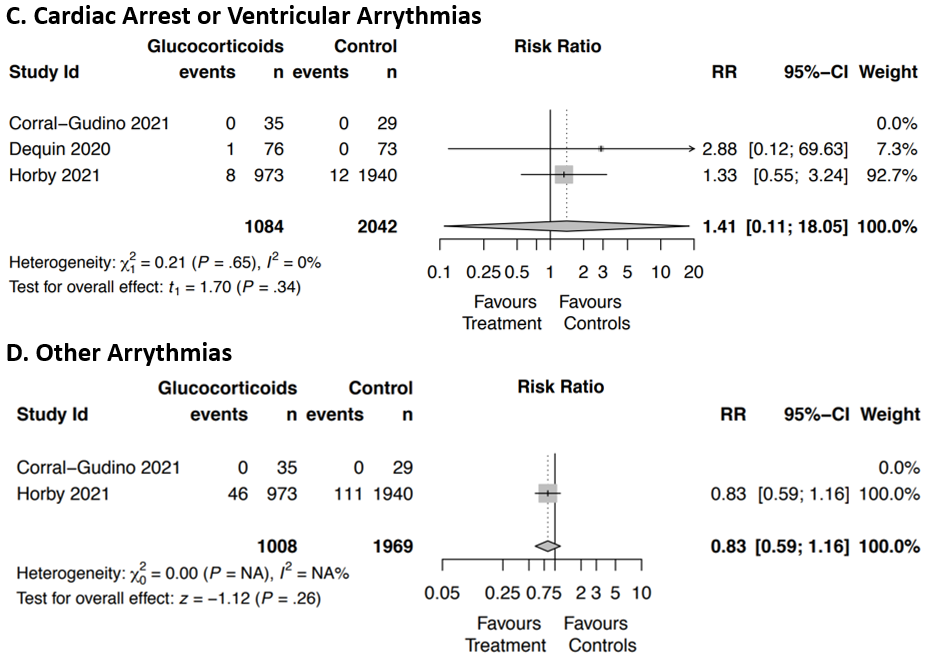


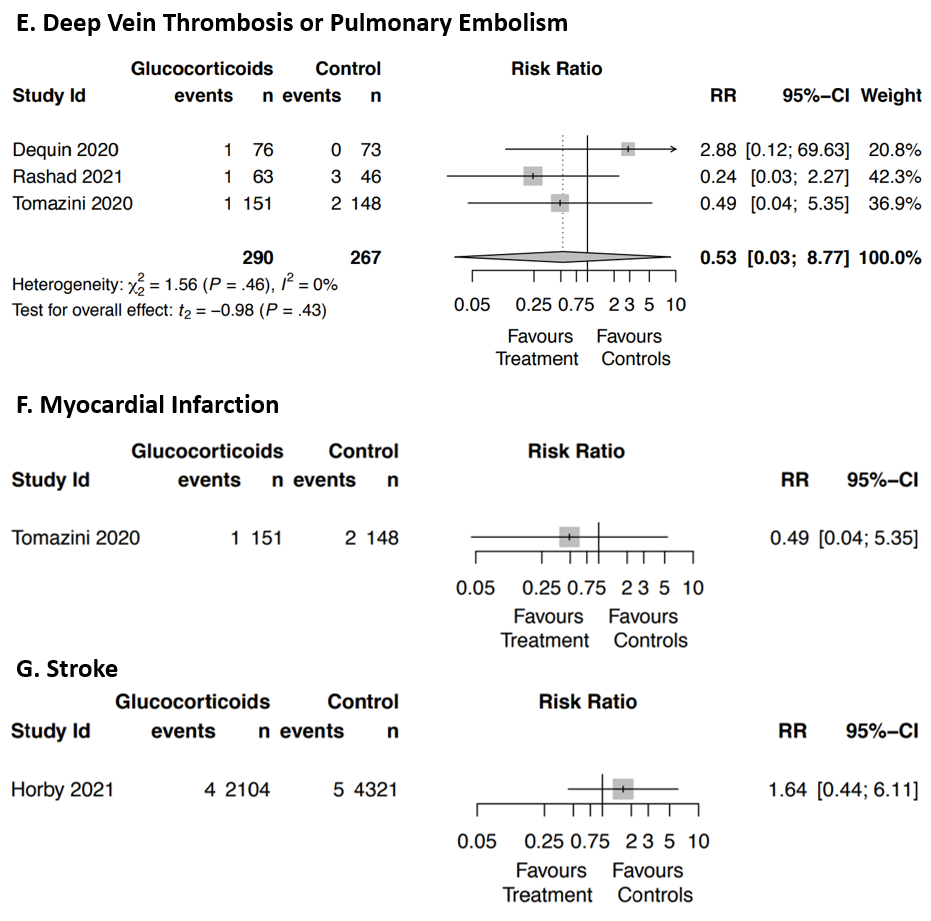


**References**

1. Pellicori P, Doolub G, Wong CM, et al. COVID-19 and its cardiovascular effects: a systematic review of prevalence studies. *Cochrane Database Syst Rev.* 2021;3:Cd013879.

2. Saand AR, Flores M, Kewan T, et al. Does inpatient hyperglycemia predict a worse outcome in COVID-19 intensive care unit patients? *J Diabetes.* 2021;13(3):253-260.

3. Mamtani M, Athavale AM, Abraham M, et al. ASSOCIATION OF HYPERGLYCEMIA WITH HOSPITAL MORTALITY IN NONDIABETIC COVID-19 PATIENTS: A COHORT STUDY. *medRxiv.* 2020:2020.2008.2031.20185157.

4. Carrasco-Sánchez FJ, López-Carmona MD, Martínez-Marcos FJ, et al. Admission hyperglycaemia as a predictor of mortality in patients hospitalized with COVID-19 regardless of diabetes status: data from the Spanish SEMI-COVID-19 Registry. *Annals of Medicine.* 2021;53(1):103-116.

5. Sardu C, D’Onofrio N, Balestrieri ML, et al. Outcomes in Patients With Hyperglycemia Affected by Covid-19: Can We Do More on Glycemic Control? *Diabetes Care.* 2020:dc200723.

6. Klonoff DC, Messler JC, Umpierrez GE, et al. Association Between Achieving Inpatient Glycemic Control and Clinical Outcomes in Hospitalized Patients With COVID-19: A Multicenter, Retrospective Hospital-Based Analysis. *Diabetes Care.* 2021;44(2):578-585.

7. Coppelli A, Giannarelli R, Aragona M, et al. Hyperglycemia at Hospital Admission Is Associated With Severity of the Prognosis in Patients Hospitalized for COVID-19: The Pisa COVID-19 Study. *Diabetes Care.* 2020;43(10):2345-2348.

8. Wang S, Ma P, Zhang S, et al. Fasting blood glucose at admission is an independent predictor for 28-day mortality in patients with COVID-19 without previous diagnosis of diabetes: a multi-centre retrospective study. *Diabetologia.* 2020;63(10):2102-2111.

9. Cano EJ, Fonseca Fuentes X, Corsini Campioli C, et al. Impact of Corticosteroids in Coronavirus Disease 2019 Outcomes: Systematic Review and Meta-analysis. *Chest.* 2021;159(3):1019-1040.

10. Chaudhuri D, Sasaki K, Karkar A, et al. Corticosteroids in COVID-19 and non-COVID-19 ARDS: a systematic review and meta-analysis. *Intensive Care Medicine.* 2021;47(5):521-537.

11. Hasan SS, Kow CS, Mustafa ZU, Merchant HA. Does methylprednisolone reduce the mortality risk in hospitalized COVID-19 patients? A meta-analysis of randomized control trials. *Expert Review of Respiratory Medicine.* 2021;15(8):1049-1055.

12. Li H, Chen C, Hu F, et al. Impact of corticosteroid therapy on outcomes of persons with SARS-CoV-2, SARS-CoV, or MERS-CoV infection: a systematic review and meta-analysis. *Leukemia.* 2020;34(6):1503-1511.

13. Li J, Liao X, Zhou Y, et al. Comparison of Associations between Glucocorticoids Treatment and Mortality in COVID-19 Patients and SARS Patients: A Systematic Review and Meta-Analysis. *Shock.* 2021;56(2):215-228.

14. Lu S, Zhou Q, Huang L, et al. Effectiveness and safety of glucocorticoids to treat COVID-19: A rapid review and meta-analysis. *Annals of Translational Medicine.* 2020;8(10):627.

15. Ma S, Xu C, Liu S, et al. Efficacy and safety of systematic corticosteroids among severe COVID-19 patients: a systematic review and meta-analysis of randomized controlled trials. *Signal Transduction and Targeted Therapy.* 2021;6(1):83.

16. Pasin L, Navalesi P, Zangrillo A, et al. Corticosteroids for Patients With Coronavirus Disease 2019 (COVID-19) With Different Disease Severity: A Meta-Analysis of Randomized Clinical Trials. *Journal of Cardiothoracic and Vascular Anesthesia.* 2021;35(2):578-584.

17. Pulakurthi YS, Pederson JM, Saravu K, et al. Corticosteroid therapy for COVID-19 A systematic review and meta-analysis of randomized controlled trials. *Medicine (United States).* 2021;100(20):e25719.

18. Sahilu T, Sheleme T, Melaku T. Severity and Mortality Associated with Steroid Use among Patients with COVID-19: A Systematic Review and Meta-Analysis. *Interdisciplinary Perspectives on Infectious Diseases.* 2021;2021 (no pagination).

19. Sarkar S, Khanna P, Soni KD. Are the steroids a blanket solution for COVID-19? A systematic review and meta-analysis. *Journal of Medical Virology.* 2021;93(3):1538-1547.

20. Sarma P, Bhattacharyya A, Kaur H, et al. Efficacy and safety of steroid therapy in COVID-19: A rapid systematic review and Meta-analysis. *Indian J Pharmacol.* 2020;52(6):535-550.

21. Sterne JAC, Murthy S, Diaz JV, et al. Association Between Administration of Systemic Corticosteroids and Mortality Among Critically Ill Patients With COVID-19: A Meta-analysis. *Jama.* 2020;324(13):1330-1341.

22. Tlayjeh H, Mhish OH, Enani MA, et al. Association of corticosteroids use and outcomes in COVID-19 patients: A systematic review and meta-analysis. *J Infect Public Health.* 2020;13(11):1652-1663.

23. van Paassen J, Vos JS, Hoekstra EM, Neumann KMI, Boot PC, Arbous SM. Corticosteroid use in COVID-19 patients: a systematic review and meta-analysis on clinical outcomes. *Critical Care.* 2020;24(1):696.

24. Wang J, Yang W, Chen P, et al. The proportion and effect of corticosteroid therapy in patients with COVID-19 infection: A systematic review and meta-analysis. *PLoS One.* 2021;16(4):e0249481.

25. Ye Z, Wang Y, Colunga-Lozano LE, et al. Efficacy and safety of corticosteroids in COVID-19 based on evidence for COVID-19, other coronavirus infections, influenza, community-acquired pneumonia and acute respiratory distress syndrome: a systematic review and meta-analysis. *Cmaj.* 2020;192(27):E755-E767.

26. Yousefifard M, Mohamed Ali K, Aghaei A, et al. Corticosteroids on the Management of Coronavirus Disease 2019 (COVID-19): A Systemic Review and Meta-Analysis. *Iran J Public Health.* 2020;49(8):1411-1421.

27. Cui Y, Sun Y, Sun J, et al. Efficacy and Safety of Corticosteroid Use in Coronavirus Disease 2019 (COVID-19): A Systematic Review and Meta-Analysis. *Infectious Diseases and Therapy.* 2021:1-17.
